# Systematic transcriptome profiling of pyroptosis related signature for predicting prognosis and immune landscape in lower grade glioma

**DOI:** 10.1186/s12885-022-09982-7

**Published:** 2022-08-13

**Authors:** Huihan Yu, Meiting Gong, Jian Qi, Chenggang Zhao, Wanxiang Niu, Suling Sun, Shuyang Li, Bo Hong, Junchao Qian, Hongzhi Wang, Xueran Chen, Zhiyou Fang

**Affiliations:** 1 School of Basic Medical Sciences, Anhui Medical University, No. 81, Meishan Road, Hefei, 230032 Anhui China; 2 Anhui Province Key Laboratory of Medical Physics and Technology; Institute of Health and Medical Technology, Hefei Institutes of Physical Science, Chinese Academy of Sciences, No. 350, Shushan Hu Road, Hefei, 230031 Anhui China; 3 Department of Laboratory Medicine, Hefei Cancer Hospital, Chinese Academy of Sciences, No. 350, Shushan Hu Road, Hefei, 230031 Anhui China

**Keywords:** Pyroptosis, Lower-grade glioma, Prognostic signature, Tumor immune microenvironment, Drug screening, Fedratinib

## Abstract

**Background:**

Pyroptosis is a programmed cell death mediated by the gasdermin superfamily, accompanied by inflammatory and immune responses. Exogenously activated pyroptosis is still not well characterized in the tumor microenvironment. Furthermore, whether pyroptosis-related genes (PRGs) in lower-grade glioma (LGG) may be used as a biomarker remains unknown.

**Methods:**

The RNA-Sequencing and clinical data of LGG patients were downloaded from publicly available databases. Bioinformatics approaches were used to analyze the relationship between PRGs and LGG patients’ prognosis, clinicopathological features, and immune status. The NMF algorithm was used to differentiate phenotypes, the LASSO regression model was used to construct prognostic signature, and GSEA was used to analyze biological functions and pathways. The expression of the signature genes was verified using qRT-PCR. In addition, the L1000FWD and CMap tools were utilized to screen potential therapeutic drugs or small molecule compounds and validate their effects in glioma cell lines using CCK-8 and colony formation assays.

**Results:**

Based on PRGs, we defined two phenotypes with different prognoses. Stepwise regression analysis was carried out to identify the 3 signature genes to construct a pyroptosis-related signature. After that, samples from the training and test cohorts were incorporated into the signature and divided by the median RiskScore value (namely, Risk-H and Risk-L). The signature shows excellent predictive LGG prognostic power in the training and validation cohorts. The prognostic signature accurately stratifies patients according to prognostic differences and has predictive value for immune cell infiltration and immune checkpoint expression. Finally, the inhibitory effect of the small molecule inhibitor fedratinib on the viability and proliferation of various glioma cells was verified using cell biology-related experiments.

**Conclusion:**

This study developed and validated a novel pyroptosis-related signature, which may assist instruct clinicians to predict the prognosis and immunological status of LGG patients more precisely. Fedratinib was found to be a small molecule inhibitor that significantly inhibits glioma cell viability and proliferation, which provides a new therapeutic strategy for gliomas.

**Supplementary Information:**

The online version contains supplementary material available at 10.1186/s12885-022-09982-7.

## Introduction

Glioma is a malignant primary brain tumor arising from cells within the central nervous system that is highly infiltrative and resistant to treatment, rendering them largely incurable [1]. Gliomas are classified by the World Health Organization (WHO) into four grades, with diffuse WHO grades II and III gliomas making up the lower-grade glioma (LGG) [2]. The clinical treatment strategies for gliomas are Postoperative radiotherapy or adjuvant temozolomide (TMZ) chemotherapy is usually recommended [3], and molecularly targeted therapies and immunotherapy have shown some positive results in recent studies [4, 5], but overall the prognosis for LGG patients remains poor [6]. Due to the significant tumor heterogeneity and cellular and molecular complexity of LGG [7, 8], it is critical to use molecular biomarkers to define tumor classification and grade. Given this, novel prognostic markers and potential therapeutic targets must be explored based on the rapid advancement of molecular analysis of tumor gene expression patterns.

Multiple modalities of programmed cell death (PCD) are thought to play a key role in the development and treatment of cancer [9]. Pyroptosis is a new modality of PCD characterized by the cleavage and activation of members of the gasdermin (GSDM) superfamily and the release of proinflammatory mediators [10]. More significantly, pyroptosis has the potential to have vast implications on cancer immunogenicity [11]. It was discovered during radiotherapy and chemotherapy that different pathways activate pyroptosis in cancers [12, 13]. The most relevant and prominent modalities of PCD in cancer, such as pyroptosis and ferroptosis, could serve as biomarkers for the early diagnosis and prognosis of cancer [14, 15]. However, the prognostic value and reliability of these pyroptosis-related biomarkers in glioma remain controversial, and the applicability of pyroptosis-related biomarkers in the complex context of pyroptosis being activated in different tumor therapies needs to be further explored. Therefore, a larger independent LGG cohort is required for further analysis and validation to provide more precise insights for clinical application.

Utilizing bioinformatics analysis, tumor genome sequence research is a vital part of cancer research [16]. It can be used to explore the molecular sub-classes of cancers [17], but it can also be used to develop biomarkers for early diagnosis, efficacy assessment, prediction, and prognosis of tumors [18, 19]. The value of biomarkers in antitumor drug development is becoming increasingly evident. The presence of polypharmacology interactions of drugs in cells should be faithfully translated into cellular phenotypic responses, and several databases have been established to analyze differential gene expression after chemical perturbations [20, 21]. Then inverse comparing the similarity in differential gene expression profiles with those after chemical perturbations, this drug prediction approach shows considerable promise [22].

Therefore, our study will explore the prognostic value and novel therapeutic targets of these genes by comparing the expression levels of PRGs in LGG and non-tumor tissues. The stability, accuracy, and predictive efficiency of pyroptosis-related signature and the nomogram model were demonstrated in the TCGA, CGGA, or Rembrandt cohorts. Further analysis of the correlation between pyroptosis and tumor immune landscape, and the use of gene expression profile to screen small molecule inhibitors, will hopefully provide potential guidance for glioma targeted therapy and immunotherapy.

## Materials and methods

### Data acquisition and preprocessing

From The Cancer Genome Atlas (TCGA) (https://portal.gdc.cancer.gov), we downloaded the RNA-Sequencing (RNA-seq) data and corresponding clinical data of LGG patients as the training cohort. Also downloaded from the TCGA were the somatic mutation data of 520 LGG samples. The normal people’s brain cortex and frontal cortex data used for different analyses were downloaded from GTEx (https://www.gtexportal.org/home/). The RNA-seq data and clinical information of LGG patients were obtained from the Chinese Glioma Genome Atlas (CGGA) (http://www.cgga.org.cn/) as the validation cohort. The validation cohort mRNAseq_693 and mRNAseq_325 were corrected for the batch effect of non-biotechnical biases by using the “sva” package in R (https://www.r-project.org/). RNA-seq derived FPKM values from the original publications have been transformed into TPM values. Also, CGGA microarray data (mRNA-array_301) from 142 LGG patients who had received radiotherapy or chemotherapy and normalized microarray format data from 133 LGG patients from the Rembrandt dataset were downloaded. The mRNA-array_301 and Rembrandt datasets were used for external validation cohort of the prognostic signature. Patients with missing clinical information and overall survival (OS) of less than 30 days were excluded from further analysis; the detailed information of these patients was listed in Table S1 (Additional file 1: Table S1). The copy number variation (CNV) data for LGG samples were obtained using the UCSC Xena browser (https://xena.ucsc.edu/). Based on previous studies, we chose 24 key regulators closely associated with pyroptosis [11, 23, 24].

### Identification of clusters using non-negative matrix factorization (NMF) algorithm

Using the “limma” package, differentially expressed pyroptotic-related genes were identified between tumor and normal tissues in the TCGA cohort. |Log2fold change(FC)| > 1 and false discovery rate (FDR) < 0.05 were set as the cutoffs for the differentially expressed genes. The NMF clustering algorithm [25] was utilized to identify clusters by extracting biological correlation coefficients and internal feature structures from the prognostic PRGs. The number of clusters was chosen based on cophenetic and residual sum of squares (rss). The number of clusters k can range from 2 to 10. During k = 2, the cluster demonstrated acceptable stability and performance in both TCGA and CGGA cohorts.

### Construction of prognostic gene signature and nomogram model

A univariate Cox proportional hazards regression model was used in conjunction with the R package “survival” to assess the expression levels of the differentially expressed genes identified. The least absolute shrinkage and selection operator (LASSO) regression model was chosen to avoid over-fitting. Finally, the minimum criteria were used to determine signature genes and their coefficients by picking the best penalty parameter λ associated with the smallest 10-fold cross-validation inside the TCGA cohort. As a result of the prognostic gene signature, the RiskScore value can be calculated for each patient with LGG as follows:

1 $$\mathrm{RiskScore}={\sum}_{\mathrm{i}=1}^{\mathrm{n}}{\upbeta}_{\mathrm{i}}\times {\upchi}_{\mathrm{i}}$$

In formula (1), βi indicates the coefficient of the gene, and χi represents the gene expression level. The area beneath the receiver operating characteristic curve (AUC) was determined using the R package “survival ROC”. The univariate and multivariate Cox regression analyses were performed on the prognostic gene signature and clinicopathological parameters (age, gender, grade, IDH mutation status, and 1p/19q codeletion status). Further, subgroups of patients were defined based on their radiotherapy and chemotherapy, and risk was quantified using prognostic gene signature. Then, using the “rms” R package [26], a nomogram model was constructed to estimate the probability of survival at 1-, 3-, and 5-years. The calibration curves were plotted to assess predicted and actual survival consistency.

### Estimation of immune cell infiltration and immune function status

We did a single sample genomic enrichment analysis (ssGSEA) of immune cell infiltration and immune function selected from previous studies. According to Charoentong et al. [27], who built The Cancer Immunome Atlas (https://tcia.at/) after characterizing the intratumoral immune landscapes and cancer antigenomes of 20 solid tumors, we chose 13 innate immune cell types and 15 adaptive immune cell types, as well as the 782 metagenes that correlate to them. Immune function-related immunomodulators from the study of Yin He et al. [28]. The ESTIMATE algorithm [29] calculated stromal, immune, ESTIMATE scores, and tumor purity. The TIMER (tumor immune estimation resource) algorithm (http://timer.comp-genomics.org/) [30] was also used to estimate immune cell infiltration. Four other algorithms, including EPIC, CIBERSORT, xCell, and quanTIseq, were applied to evaluate macrophage infiltration. The “clusterprofiler” package [31] was used to run enrichment analysis for Gene Ontology (GO) and Kyoto Encyclopedia of Genes and Genomes (KEGG) [32].

### Immunotherapy prediction

We expanded on 27 immune checkpoints already identified in prior studies, including the B7-CD28 family (PD-1, PD-L1, PD-L2, CTLA4, B3-H7, B7-H5, ICOS, ICOSLG, CD28H, and B7-H4) [33], the TNF superfamily (BTLA, CD27, CD40, CD40LG, CD70, GITR, OX40, 4-1BB, and HVEM) [34]. Several other molecules (TIM3, IDO1, LAG3, and TIGIT) were further explored. We validated immunotherapy responses using two immunotherapy cohorts, including a metastatic melanoma cohort treated with pembrolizumab, an anti-PD-1 antibody (GSE78220 cohort) [35], and an advanced urothelial cancer cohort treated with atezolizumab, an anti-PD-L1 antibody (IMvigor210 cohort) [36]. FPKM values of gene expression data in the dataset have been transformed into TPM values. The TIDE algorithm (http://tide.dfci.harvard.edu/) [37] can model the two primary pathways of tumor immune evasion, T-cell dysfunction, and T-cell rejection. Thus, we generated TIDE scores for LGG patients to estimate the immunotherapy response of risk subgroups.

### Potential drugs or small molecule compounds prediction

To perform gene set enrichment analysis (GSEA) on samples from the Risk-H and Risk-L groups, we downloaded “c2.cp.reactome.v7.5.1.symbols,” and “c2.cp.kegg.v7.5.1.symbols” from the MSigDB database (http://www.gsea-msigdb.org/gsea/downloads.jsp). We reverse-screened for medicines or small chemical compounds against differential genes in the Risk-H and Risk-L groups using the L1000 fireworks display (L1000FWD) web program (http://amp.pharm.mssm.edu/l1000fwd/) [20]. The following analysis comprised drugs or small molecule compounds with a similarity score < − 0.1. The connectivity map (CMap) (https://clue.io/) [21] is a compilation of data on the transcriptional expression of the whole genome derived from human cell lines treated with various medications. The functional connections between medications, genes, and illnesses were then discovered using pattern-matching algorithms and frequently gene expression changes features. Candidate compounds with connectivity scores<− 0.55 and *P* < 0.05 were identified as potential therapeutic candidates.

### Cell lines and chemical reagents

Human glioma cell lines (T98G, SW1088, SW1783, A172, and LN18) and normal people glial cell line HEB were obtained from the American Type Culture Collection (ATCC) from 2016 to 2018 and characterized with isozyme detection and DNA fingerprinting. A low-passage aliquot of each line was thawed every 3 to 4 months to be used in our experiments. This study used all mycoplasma-free cell lines that were validated regularly. Glioma cell lines were cultured in DMEM supplemented with 1% (100×) streptomycin/penicillin and 10% FBS. Cells were maintained at 37 °C in a humidified atmosphere of 5% CO2. The EMBL-EBI Expression Atlas (https://www.ebi.ac.uk/gxa/home) was used to obtain RNA-seq data for signature genes in glioma cell lines.

Fedratinib (TG-101348) was purchased from MedChemExpress (Monmouth Junction, NJ, USA).

### RNA extraction and real-time quantitative PCR (qRT-PCR)

Total RNAs from cell lines were extracted by RNA preparation kit (TransGen Biotech, Beijing). Subsequently, using the HiScript II Q RT SuperMix for qPCR (+gDNA wiper) (Vazyme Biotech, Nanjing), the RNA was reversely transcribed into cDNA, and qRT-PCR analyses were quantified with ChamQ Universal SYBR qPCR Master Mix (Vazyme Biotech, Nanjing). Utilizing GAPDH as the internal control, every sample was prepared in triplicate. The primers used were as follows: human GAPDH(F) 5′-GTCTCCTCTGACTTCAACAGCG-3′; human GAPDH(R) 5′-ACCACCCTGTTGCTGTAGCCAA-3′; human CASP1(F) 5′-ATATGCCTGTTCCTGTGATGTG-3′; human CASP1(R) 5′-TGTCAAAGTCACTCTTTCAGTGG-3′; human CASP3(F) 5′-GATTATCCTGAGATGGGTTTATG-3′; human CASP3(R) 5′-GAATGTTTCCCTGAGGTTTGC-3′; human CASP9(F) 5′-AGTAACCCCGAGCCAGATGC-3′; human CASP9(R) 5′-GTCCCTCCAGGAAACAAAACC-3′. Furthermore, we confirmed the protein expression level for the pyroptosis-associated prognostic gene using immunohistochemistry data publicly available at the Human Protein Atlas (http://www.proteinatlas.org/).

### Cell viability assay

Glioma cells were seeded in 96-well plates at 6000 cells per well and incubated overnight. The cells were treated with different concentrations of fedratinib (1, 2, 4, 6, 8, 10, and 25 μM) followed by a 24- and 48-hours incubation. Next, the cells were treated with WST-8 from CCK-8 (NCM Biotech, Suzhou, China) for 0.5-1 hours; then their viability was measured by detecting the absorbance at OD 450 nm.

### Colony formation assay

In a 6-well plate, 3000 glioma cells were plated in triplicate and incubated overnight, then grown for 10 days in a growth medium with fedratinib (0, 0.4, 0.6, 0.8, and 1 μM). We then washed the cells three times with PBS, fixed them in cold methanol for 20 minutes, and washed and stored them. Fixed cell colonies were visualized by incubating the cells with 0.5% (w/v) crystal violet for 0.5 hours. Extra crystal violet was removed by washing with PBS. Visible colonies formed by glioma cell growth were identified using the ColonyArea plugin [38] in ImageJ version 2.3.0. Run the “Colony measurer” tool to measure the colony area and intensity percentages. There is a parameter called ‘colony area percentage,’ which refers to the percentage of a well’s surface covered by cells, and a second parameter, the intensity weighted area percentage, called ‘colony intensity percent.’ The colony area percentage may be more reflective of cell survival, while colony intensity percentage reports on the ability of the cells to grow densely.

### Statistical analysis and visualization

R version 4.1.1 and GraphPad Prism version 9.0 software were used for statistical analysis and visualization. The Kaplan–Meier curve was used to determine the statistical significance of the survival rates between different risk groups, which were then contrasted utilizing the log-rank test. The Wilcox test was used to analyze non-normally distributed continuous variables. A one-way ANOVA analysis was utilized to evaluate the differences between more than two groups. For the comparison of categorical variables, Pearson’s chi-square tests were used. |Log2FC| > 1.3 and FDR < 0.05 were set as the cutoffs for the differentially expressed genes between Risk-H and Risk-L groups. *P* < 0.05 was deemed statistically significant unless otherwise stated. GSEA was performed using GSEA version 4.2.3 software (http://www.gsea-msigdb.org/gsea/index.jsp).

## Results

### Mining of differentially expressed PRGs and cluster analysis

**Fig. 1:**
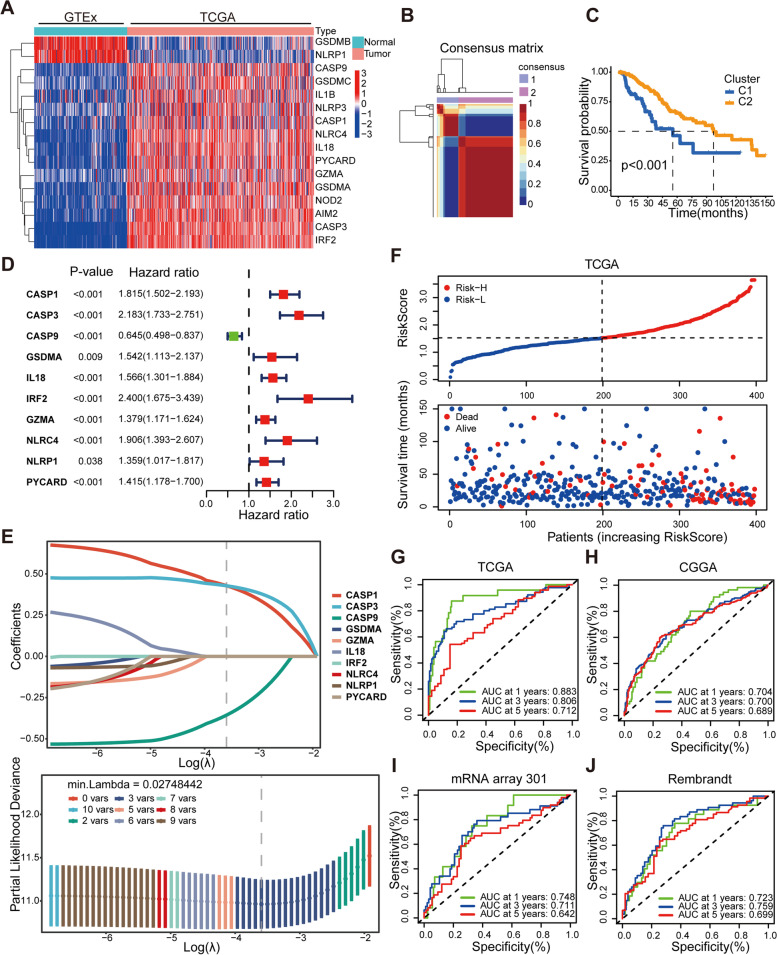
The process of constructing PRGs-related clusters and prognostic signatures. **A** Unsupervised clustering of differential expression genes between tumor patients and normal person in the TCGA and GTEx cohorts. Heatmap show 16 differential gene expression levels with *P* < 0.001. Red indicates up-regulation of gene expression, and blue indicates down-regulation. **B** During clusters k = 2, consensus map of NMF clustering in TCGA training cohort. **C** The Kaplan-Meier curves for the two clusters based on TCGA cohort. **D** The hazard ratios (HR) and 95% confidence intervals (CI) calculated by univariate Cox regression are shown. **E** Each independent variable’s changing trajectory. A horizontal axis represents the logarithm of lambda, and a vertical axis represents its coefficient. Increasing lambda increases the number of independent variable coefficients that tend to 0. Confidence intervals for each lambda. When the lambda is 0.02748, an optimal model is acquired. **F** The scattergrams of the RiskScore value (up) and survival status (down) of LGG patients in the TCGA cohorts. **G-J** The 1-, 3- or 5-year OS predicted ROC curves of a prognostic signature in the TCGA, CGGA, mRNA array 301, and Rembrandt cohorts. The 1-, 3- or 5-year AUC is shown in the Figure.

First, based on the data of somatic mutations and copy number variation in LGG patients, 24 pyroptosis regulators were analyzed. Mutations in IDH1 (78%), TP53 (47%), and ATRX (37%) were found in 520 LGG patients, which is consistent with the results of previous studies [7], but somatic mutations in pyroptosis regulators were almost absent in all LGG molecular subtypes (Additional file 2: Fig. S1A). We found certain levels of DNA fragment copy number deletion in the following genes: GSDMD, GZMB, NLRP3, CASP3 and CASP9, IRF1 and IRF2. However, the overall deletion frequency was less than 8%. GSDMC and GSDMD exhibited copy number amplifications of about 6-8%. (Additional file 2: Fig. S1B). Protein-protein interaction network analysis (PPI) analysis was used to identify hub genes, and correlation analysis was performed to determine the expression correlation between the pyroptosis regulators (Additional file 2: Figs. S1C, D). The WHO classification system confirmed the significant correlations between grades and expressions of most pyroptosis regulators by quantitative analyses in the TCGA and CGGA cohorts (Additional file 2: Figs. S1E, F). As the grade increased, the expression of CASP1, CASP3, CASP4, CASP5, CASP8, IL18, IRF1, GZMA, GZMB, NLRC4, NOD1, NOD2, and GSDMD increased, while the expression of CASP9 decreased. Moreover, we screened for differentially expressed pyroptosis regulators between LGG and the normal cerebral cortex. Based on the results of differential gene analysis from the TCGA cohort, 16 genes with differential expression were selected, among which CASP1, CASP3, CASP9, IL1B, IL18, GZMA, NOD2, AIM2, IRF2, NLRP3, NLRC4, PYCARD, GSDMA, and GSDMC were upregulated and NLRP1 and GSDMB being downregulation (Fig. 1A). In comparing the TCGA cohort and the GTEx cohort, 16 differentially expressed PRGs were obtained. Out of these genes, two clusters were established using NMF as described in materials and methods (Additional file 2: Fig. S2A). Samples from either the TCGA or CGGA cohorts were shown to be divided into two clusters: C1 and C2 (Fig. 1B and Additional file 2: Fig. S2B). The Kaplan-Meier curves revealed a statistically significant difference in OS between the two clusters, with the C1 subgroup showing a significantly better prognosis (Fig. 1C and Additional file 2: Fig. S2C). Based on previous studies [7], we divided patients in the TCGA and CGGA cohorts into three molecular subtypes: IDH mutant with 1p/19q codeletion, IDH mutant without 1p/19q codeletion, and IDH wild-type. LGGs with both an IDH mutation and deletion of chromosome arms 1p and 19q (1p/19q) are associated with longer survival than diffuse gliomas without these alterations. Most LGGs without an IDH mutation were molecularly and clinically like glioblastoma. Among the two pyroptosis-associated clusters, more LGG patients with IDH wild-type were found in the C1 subgroup, while more LGG patients with IDH mutant and 1p/19q codeletion were found in the C2 subgroup (Additional file 2: Fig. S2D).

### Construction of the signature to predict prognosis for LGG patients

In the TCGA cohort, using univariate Cox analysis, 9 risk factors (CASP1, CASP3, IL18, IRF2, GZMA, NLRC4, NLRP1, PYCARD, and GSDMA) and protective factor CASP9 associated with the prognosis of LGG patients were identified for further analysis (Fig. 1D). Additionally, these 10 genes were refined using LASSO regression to reduce the number of signature genes recruited into this prognostic signature: RiskScore = (0.428519*mRNA Expression value of CASP1) + (0.427944*mRNA Expression value of CASP3) + (− 0.356325*mRNA Expression value of CASP9). The R package “glmnet” was used to do LASSO regression analysis in this circumstance. The variation trajectory of each independent variable was assessed, and it was discovered that the majority of independent parameters had coefficients close to 0 with the gradual lambda increase (Fig. 1E). According to the confidence intervals for each lambda, the optimum model was obtained when the lambda was 0.02748. As a result, this model was chosen as the final prognostic gene signature. Each LGG patient’s RiskScore value was generated using the prognostic gene signature. Risk stratification was performed based on the median RiskScore value, and LGG patients were divided into the Risk-H and Risk-L groups (Fig. 1F and Additional file 2: Fig. S2E). Additionally, time-dependent receiver operating characteristic (ROC) analysis revealed favorable prognostic predictions (1-year AUC = 0.883, 3-year AUC = 0.806, and 5-year AUC = 0.712) (Fig. 1G). The ROC analysis in the CGGA validation cohort also showed promising prognostic predictions (1-year AUC = 0.704, 3-year AUC = 0.700, and 5-year AUC = 0.689) (Fig. 1H). Principal component analysis (PCA) revealed a clear division of patients with different risks into two subgroups in the TCGA and CGGA cohorts (Additional file 2: Figs. S2F, G). In both the mRNA array 301 and Rembrandt tissue microarray external validation sets, the ROC analysis showed equally excellent predictive efficiency (Fig. 1I, J), and the Kaplan-Meier curves showed significant differences in OS for patients in different risk stratification (Additional file 2: Figs. S2H, I). In addition, we used heatmaps to visualize the expression of 24 key pyroptosis regulators in risk stratification (Additional file 2: Fig. S2J). Overall, the results revealed a correlation between PRGs expression and the OS of LGG patients, thus suggesting that PRGs may reflect certain characteristics in tumor patients.

### Correlation analysis of the RiskScore value with clinical features

**Fig. 2:**
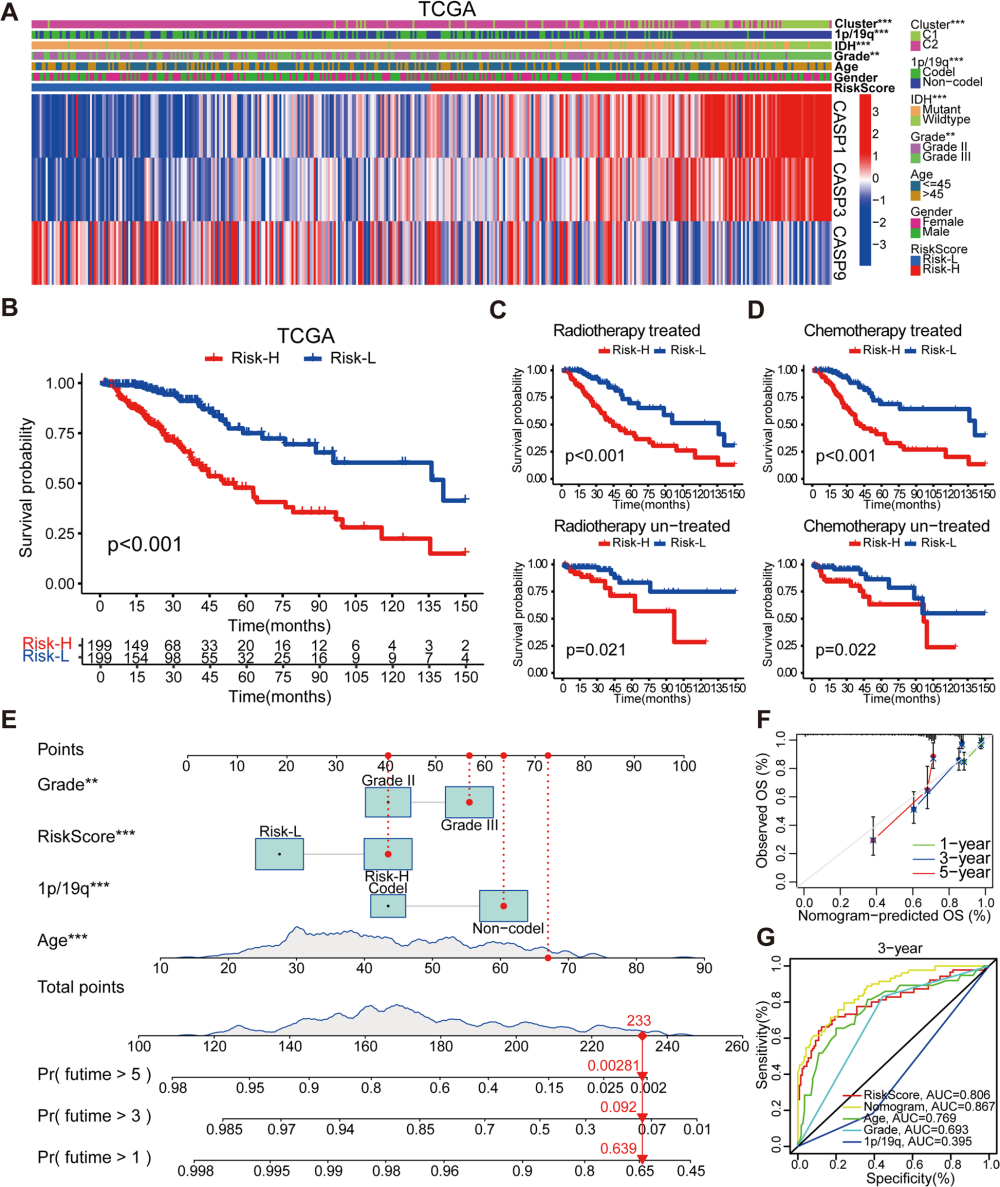
Relationship between the RiskScore value and clinical characteristics and construction of nomogram model. **A** The heatmap shows the expression levels of the 3 signature genes in the Risk-H and Risk-L groups. The distribution of clinical characteristics and C1/2 subgroups was compared between the Risk-H and Risk-L groups in the TCGA cohort. **B** The Kaplan–Meier survival curve of the prognostic signature predicts the Risk-H and Risk-L groups in the TCGA cohort. **C-D** The Kaplan-Meier survival curves predict the OS of the LGG patients receiving different treatment strategies in the TCGA cohorts. **E** The nomogram model predicts the patient’s OS. Each variable’s points on the point scale are totaled. Finally, beneath the total points, the probability of 1-, 3-, or 5-year survival is projected on the scales below. **F** Calibration curves of the nomogram model in terms of the agreement between predicted and observed 1-, 3- and 5-year outcomes. **G** ROC curves showed the predictive efficiency of the RiskScore value, nomogram model, grade, 1p/19q codeletion status, and age on the 3-year survival rate. *P < 0.05, **P < 0.01, and ***P < 0.001

Considering the critical biological functions of 3 signature genes in tumorigenesis and development, we systematically investigated the relationships between each gene and the clinical parameters of LGG, including cluster, age, gender, grade, IDH mutation status, and 1p/19q codeletion status (Fig. 2A and Additional file 2: Fig. S3A). Also, we found that the C1 subgroup was enriched for patients with higher WHO grades, IDH wild-type, and 1p/19q non-codeletion status. The correlation between the RiskScore value and statistically significant clinicopathological characteristics in the TCGA and CGGA cohorts was quantitatively analyzed. Correlation analysis revealed a significant association between high RiskScore value and higher WHO grades, IDH wild-type, 1p/19q non-codeletion status, and the C1 subgroup (Additional file 2: Figs. S3B, C). Following that, we proved the RiskScore value’s validity in predicting OS in two risk subgroups of LGG patients. The Kaplan-Meier curves in the TCGA and CGGA cohorts indicated that the Risk-H group had a more transitory OS (Fig. 2B and Additional file 2: Fig. S3D). We then extracted radiotherapy and chemotherapy treatment status to determine whether the RiskScore value could be used to identify the outcomes of various therapies in LGG patients. As expected, we discovered that the RiskScore value was an excellent predictor of OS regardless of whether LGG patients received radiotherapy or chemotherapy (Fig. 2C, D). The same results are also shown in the CGGA cohort (Additional file 2: Figs. S3E, F).

In addition, we utilized univariate and multivariate Cox regression to examine the relationships between the RiskScore value and OS in LGG and to uncover independent prognostic factors. Complete analysis of the TCGA and CGGA cohorts showed that grade, age, 1p/19q codeletion status, and RiskScore value were independent risk factors (Additional file 3: Table S2). Therefore, the nomogram model based on multivariate cox regression was constructed to predict 1-, 3-, and 5-year survival (Fig. 2E). The calibration curve depicts the predicted values of survival probability at 1-, 3-, and 5-year, and the results indicate that the prediction efficiency of our nomogram model is close to the best value in the prediction (Fig. 2F). Finally, using ROC analysis, the predictive validity of the nomogram model was also confirmed. The AUC of the nomogram model for 3-year OS prediction was 0.867, exceeding the predictive efficiency of independent prognostic factors (Fig. 2G).

### Characterization of the immune infiltration landscape based on risk stratification

**Fig. 3:**
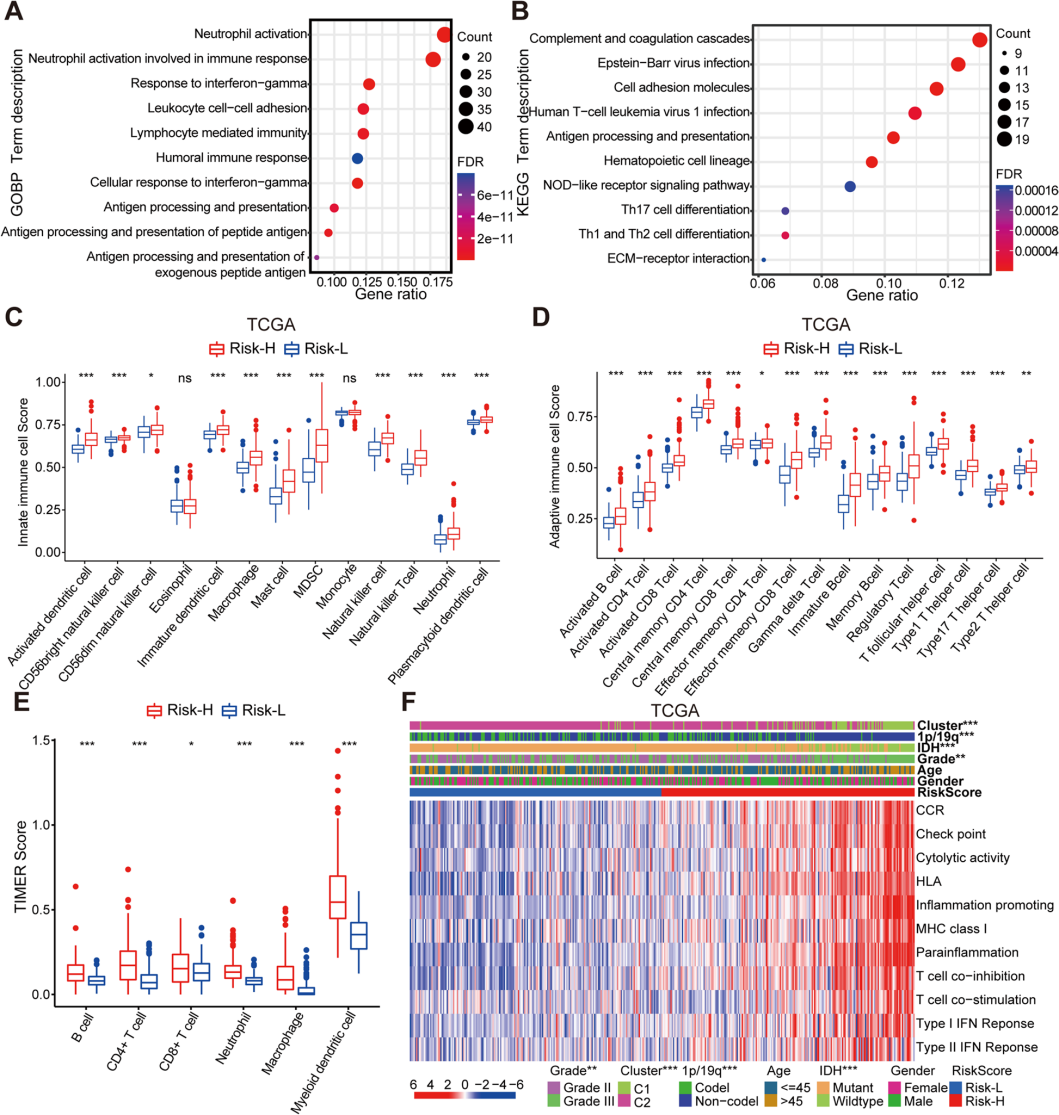
Functional annotation and analysis of immunological characteristics based on risk subgroups. **A-B** Using GO terms of biological processes and KEGG pathway analysis, bubble plots show 10 immune-related functional annotations of differentially expressed genes between the Risk-H and Risk-L groups. **C-D** Comparison of the abundance of innate and adaptive immune cell infiltration between two risk subgroups in the TCGA cohort. **E** The correlation between the RiskScore value and 6 immune cells was estimated by the TIMER algorithm. **F** The abundance of 11 immune functions by ssGSEA algorithm in the Risk-H and Risk-L groups in the TCGA cohort. *P < 0.05, **P < 0.01, and ***P < 0.001

To elucidate the biological functions and potential pathways, we used differentially expressed genes in the Risk-H and Risk-L groups to conduct GO and KEGG enrichment analyses. The results of the GO analysis revealed an enrichment of immune-related biological process terms, including neutrophil activation, response to interferon-gamma, neutrophil activation involved in immune response, antigen processing and presentation of peptide antigen, lymphocyte-mediated immunity, leukocyte cell-cell adhesion, antigen processing and presentation, and humoral immune response (Fig. 3A). Immune-related pathways such as the Complement and coagulation cascades, Antigen processing and presentation, cell adhesion molecules, Epstein-Barr virus infection, Human T-cell leukemia virus 1 infection, Th1 and Th2 cell differentiation, Th17 cell differentiation, NOD-like receptor signaling pathway, and ECM-receptor interaction are included in the KEGG terms (Fig. 3B). This suggests that most genes are enriched by different immune-related signaling pathways and biological processes.

Characterizing the immune infiltration landscape is vital for studying the tumor-immune interaction. Therefore, we analyzed the relationship between the RiskScore value and tumor immune cell infiltration and immune function using the ssGSEA enrichment score. Our analysis of the innate immune cells in TCGA and CGGA cohorts suggests that in addition to eosinophils, monocytes, and CD56dim natural killer cells, other immune cells such as macrophages, neutrophils, natural killer cells, myeloid-derived suppressor cells (MDSC), mast cells, and different dendritic cells were upregulated in the Risk-H group (Fig. 3C and Additional file 2: Fig. S4A). Additionally, 15 adaptive immune cells, particularly a variety of T cells, also showed abundant recruitment in the Risk-H group (Fig. 3D and Additional file 2: Fig. S4B). The TIMER web server is a powerful tool for analyzing immune cells that have infiltrated tumors. First, we retrieved the TIMER Scores for 6 critical immune cells found in patients with LGG in the TCGA cohort, including B cells, CD4+ T cells, CD8+ T cells, macrophages, neutrophils, and myeloid dendritic cells. As expected, significant recruitment of immune cells also occurred in the Risk-H group (Fig. 3E). After that, we further explored the correlation of the expression of 3 signature genes with immune infiltration levels in LGG. We collated scatter plots showing the tumor purity-adjusted spearman’s rho. CASP1 and CASP3 were all positively correlated with infiltration of B cells, CD4+ T cells, neutrophils, macrophages, and dendritic cells but negatively correlated with the infiltration of CD8+ T cells. CASP9 expression showed a negative or low correlation with the level of immune infiltration (Additional file 2: Fig. S4C). Tumor-associated macrophages are critical players in the immune interaction between the tumor and the host and in the progression of cancer [39]. This study analyzed macrophage infiltrates estimation values using four different immune infiltration analysis algorithms. Interestingly, macrophages or M1 and M2 subtypes classified according to macrophage polarization status showed recruitment in the Risk-H group (Additional file 2: Fig. S4D). 

Finally, in both the TCGA and CGGA cohorts, immune function or related biological processes were enriched in the Risk-H group, including CCR, checkpoint, cytolytic activity, HLA, inflammation promoting, MHC class I, parainflammation, T-cell co-inhibition, T-cell co-stimulation, type I interferon response, and type II interferon response (Fig. 3F and Additional file 2: Fig. S4E). These study results indicate the presence of a more aggressive state of immune activation in the Risk-H group differentiated by prognostic signature.

### Analysis of the immunotherapy-related characteristics

**Fig. 4:**
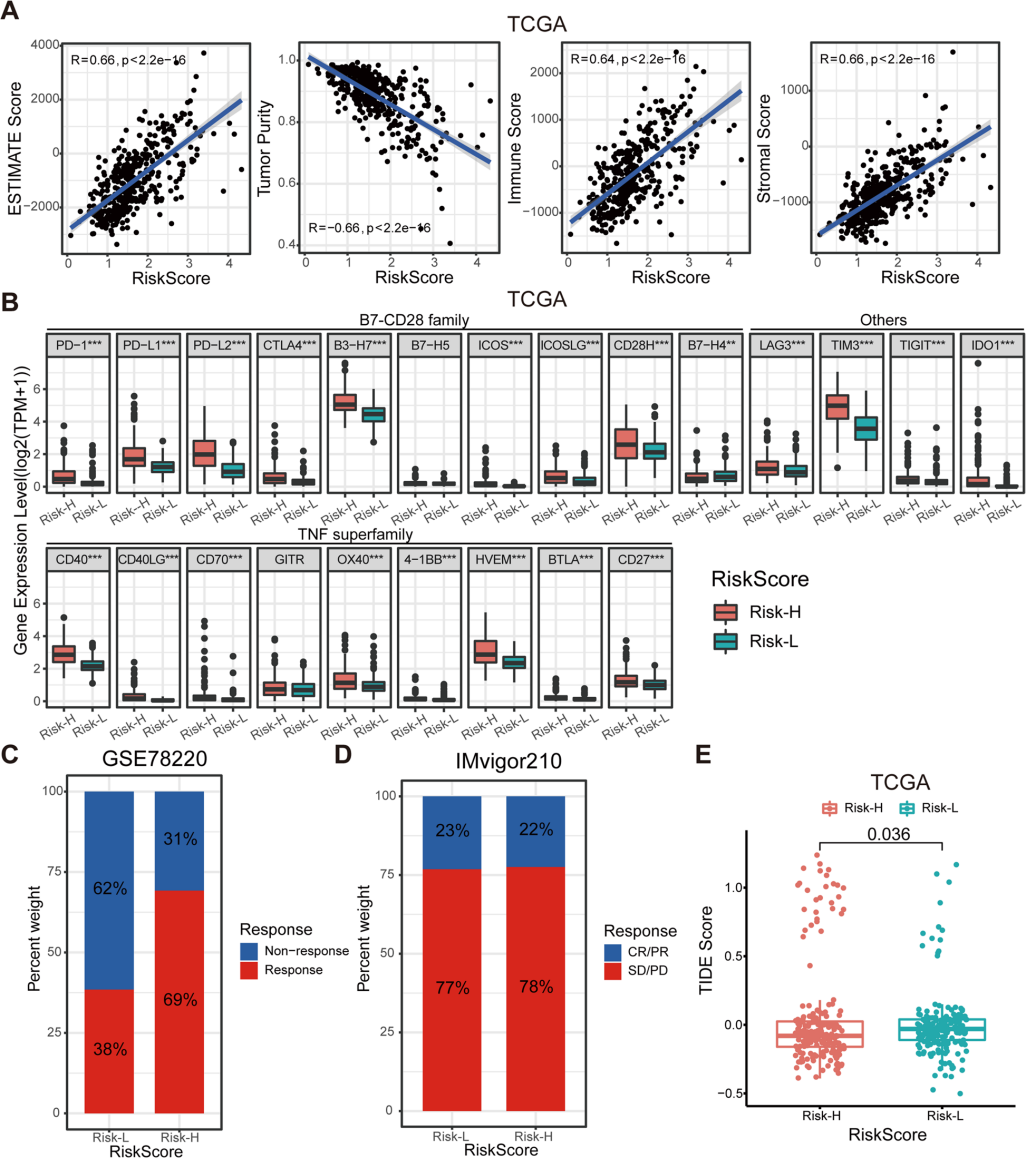
Different immune checkpoint profiles and immunotherapeutic responses of risk subgroups. **A** The association between RiskScore value and stromal score, immune score, ESTIMATE score, and tumor purity in the TCGA cohort. **B** The expression levels of 23 immune checkpoint profiles in LGG with different risk subgroups in the TCGA cohort. **C** Bar plot showing different responses to anti-PD-1 immunotherapy among 27 patients with melanoma between two risk subgroups. **D** Bar plot showing different responses to anti-PD-L1 immunotherapy among 298 patients with urothelial carcinoma between two risk subgroups. **E** Comparison of TIDE scores between Risk-H and Risk-L groups. *P < 0.05, **P < 0.01, and ***P < 0.001

We used the ESTIMATE algorithm to examine the association between the RiskScore value and immunological microenvironment. Tumor purity is a major confounding factor in the analysis, with a high RiskScore value indicating low tumor purity. The RiskScore value was strongly positively connected with the stromal, immunological, and ESTIMATE scores (Fig. 4A and Additional file 2: Fig. S5A), indicating that patients with higher immune amounts of stromal cells had higher RiskScore value.

Pyroptosis has the potential to transform the tumor microenvironment from “cold” to “hot,” and its application in cancer treatment, particularly in conjunction with immunotherapy, is promising [40]. The clinical immunotherapy strategies are primarily determined by the expression status of immune checkpoint molecules. Therefore, we explored the association between the RiskScore value and immune checkpoint. In the TCGA and CGGA cohorts, the expression of most of the immune checkpoints was significantly upregulated in the Risk-H group, especially PD-1, PD-L1, PD-L2, CTLA-4, B3-H7, CD40, and TIM-3 (Fig. 4B and Additional file 2: Fig. S5B). These upregulated immune checkpoints could be potential therapeutic targets. Most immunotherapy agents target and inhibit immune checkpoints, allowing the immune system to overcome immunity escape achieved by overexpression of immune checkpoints and enable neoantigen cancer cells to be preyed upon by the immune system. Our RiskScore value algorithm was applied to the GSE78220 and IMvigor210 cohorts to measure the predictive ability of RiskScore value in immunotherapy. Among 27 patients with melanoma treated with the anti-PD-1 drug pembrolizumab, a higher response rate was observed in the Risk-H group (Fig. 4C), demonstrating a stronger immunotherapy sensitivity in the Risk-H group. However, in the IMvigor210 cohort of patients with urothelial carcinoma treated with the anti-PD-L1 antibody atezolizumab, the response rates to immunotherapy were almost identical in the Risk-H and Risk-L groups (Fig. 4D). Next, The TIDE algorithm was used to compute TIDE values for each LGG patient in the TCGA cohort. A low TIDE value indicates a higher chance of benefiting from immune checkpoint blockade, and our research found that patients in the Risk-H group had lower TIDE values than those in the Risk-L group (Fig. 4E).

### Validation of expression in glioma cell lines and human tissues

**Fig. 5:**
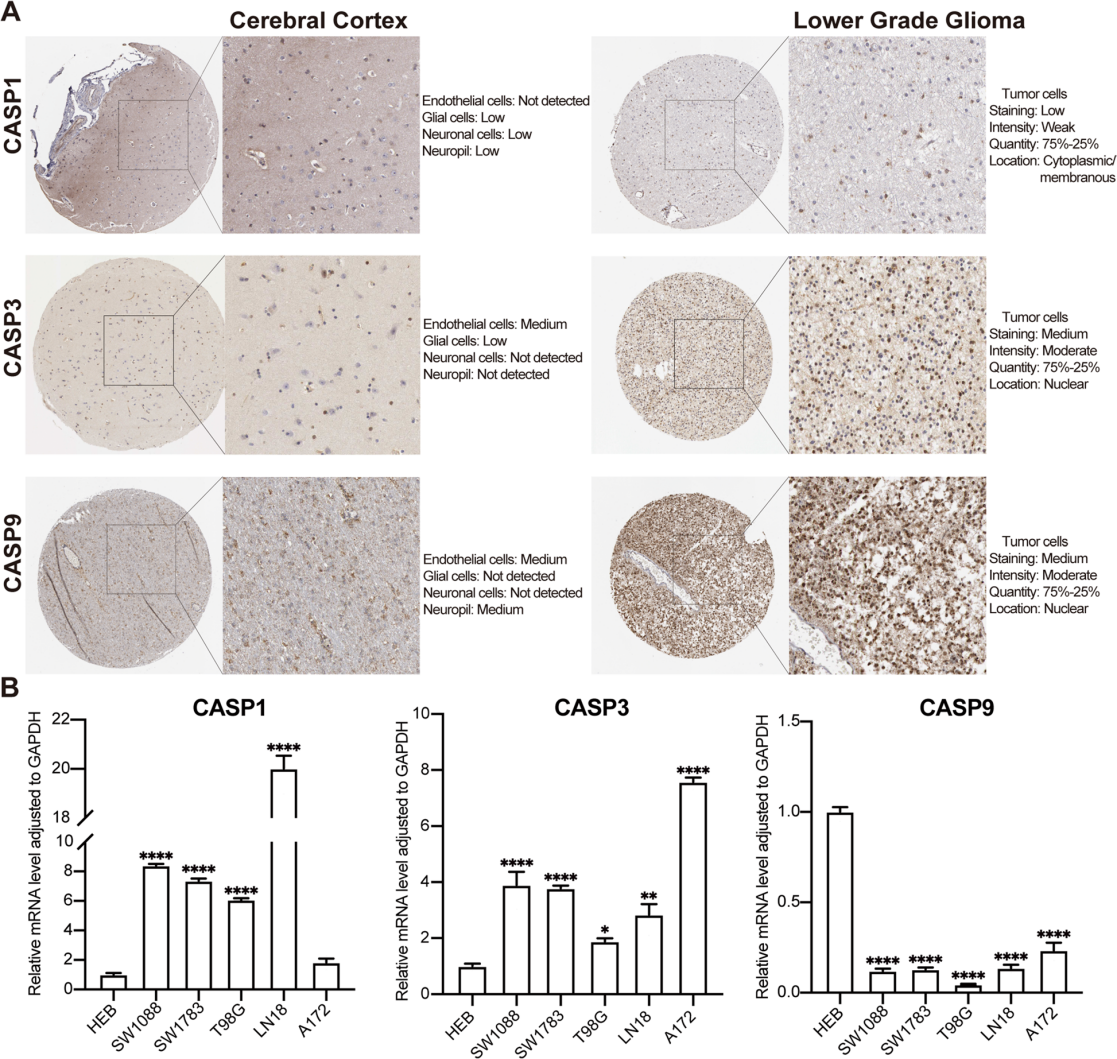
The expression verification of 3 signature genes in LGG. **A** The immunohistochemistry of 3 signature genes in the HPA database. **B** Histogram of different transcript levels of CASP1, CASP3, and CASP9 in glioma cell lines and normal people glial cell line HEB. *P < 0.05, **P < 0.01, and ****P < 0.0001

To validate the expression levels of the 3 signature genes in glioma, we first collated immunohistochemical results in the Human Protein Atlas database. We detected protein expressions of the normal people cerebral cortex and LGG. The comparison showed that CASP3 and CASP9 were a higher expression in LGG, and CASP1 staining was consistent in tumor cells and glial cells (Fig. 5A). Next, its mRNA expression level in glioma cell lines (SW1783, SW1088, T98G, LN18, and A172) and HEB cell by qRT-PCR indicated that CASP1 and CASP3 expression overall showed overall an upward trend in glioma cell lines (Fig. 5B). However, CASP9 showed lower expression levels in the 3 glioma cell lines, which we consider might be due to variations in the microenvironment between tumor cells cultured in vitro and tumor tissues (Fig. 5B).

### Screening of fedratinib as a potential therapeutic agent for glioma

**Fig. 6:**
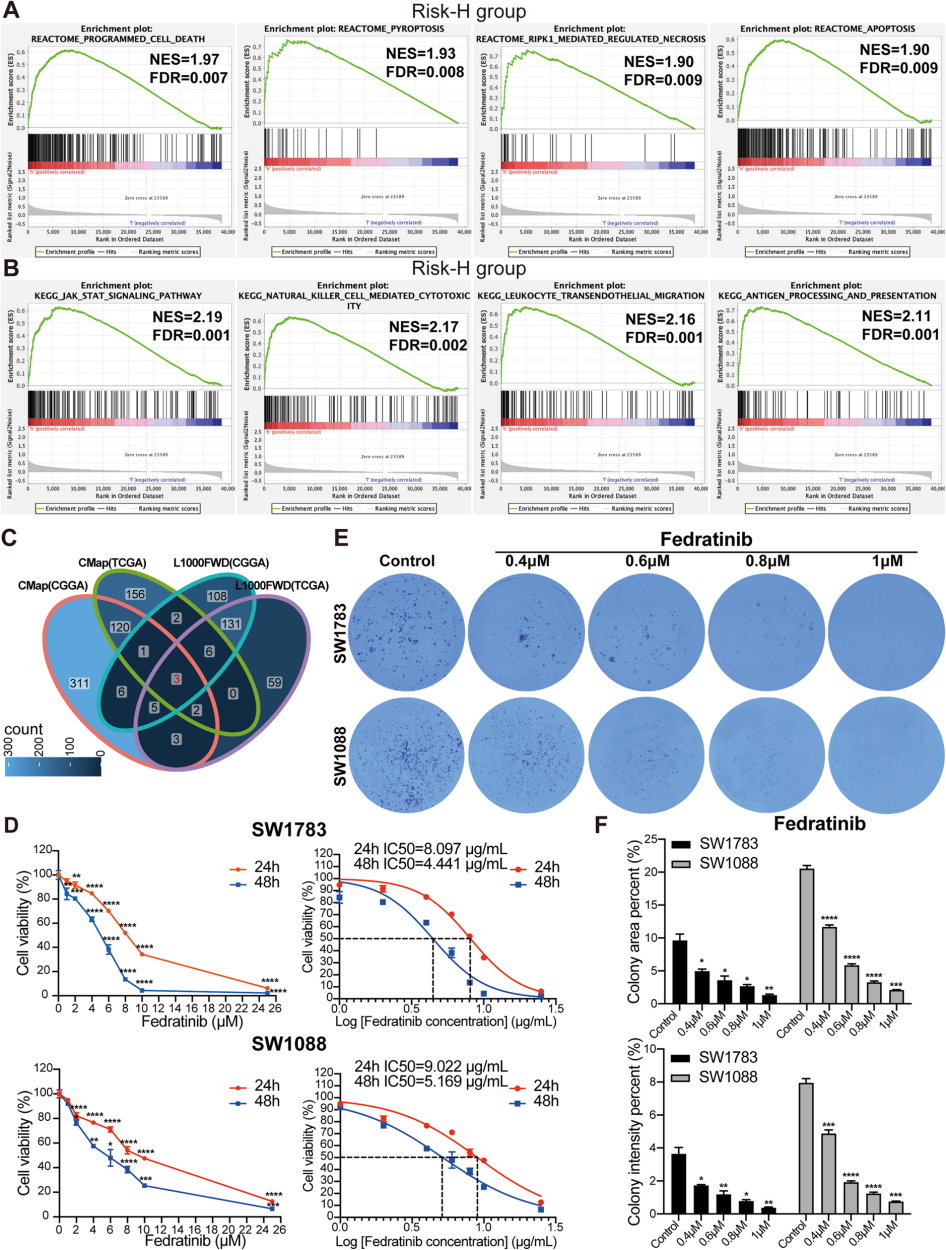
Fedratinib’s impact on the viability and proliferation of LGG cells. **A** The GSEA of biological pathways indicated the PCD pathway, pyroptosis pathway, apoptosis pathway, and RIPK1 mediated regulated necrosis pathway in the Risk-H group. **B** The GSEA of signaling cascades indicated JAK-STAT signaling pathway, natural killer cell mediated cytotoxicity pathway, leukocyte transendothelial migration pathway, and antigen processing and presentation in the Risk-H group. **C** The Venn plot shows the intersection of the four drug screening results, with the intersection showing three drugs fedratinib, XMD-892, and MMPX. **D** An CCK-8 assay was used to evaluate the viability of SW1783 and SW1088 cells under different concentrations of fedratinib (1, 2, 4, 6, 8, 10, and 25 μM). Cells were treated with the same drug dose for 24 and 48 hours, respectively. GraphPad Prism was used to analyze and visualize the data from the LGG cells viability assay and the IC50 of fedratinib. *P < 0.05, **P < 0.01, ***P < 0.001, and ****P < 0.0001 vs. control group. **E** Colony formation assay was performed to detect the colony formation ability of LGG cells after fedratinib treatment. **F** Quantitative analysis of colony formation formed by LGG cells was performed using ImageJ, followed by visualization performed using GraphPad Prism. *P < 0.05, and **P < 0.01 vs. control group

In the development of new anti-tumor drugs, biomarkers play a critical role. Rational application of biomarkers improves the efficiency of anti-tumor drug development. The GSEA was applied on samples within the Risk-H and Risk-L groups to demonstrate the possible over-representation of signaling cascades. As expected, the biological pathways in Reactome showed enrichment in programmed cell death and pyroptosis pathway. Interestingly, the Risk-H group also showed enrichment for apoptosis and RIPK1 mediated regulated necrosis pathway, which we consider is linked to the extensive crosstalk of key features in the three PCD pathways (Fig. 6A). Furthermore, the relationship between KEGG functional enrichment and RiskScore value was investigated by selecting the four major pathways in the Risk-H group with the highest normalized enrichment scores (NES). In the analysis, the JAK-STAT signaling pathway showed the highest NES score. The other three terms were associated with immune pathways, including natural killer cell, mediated cytotoxicity, leukocyte transendothelial migration, and antigen processing and presentation (Fig. 6B).

Subsequently, we screened anti-tumor drugs or small molecule compounds using differentially expressed genes based on risk stratification. We evaluated the overlap of potential drugs or small molecule compounds derived from differentially expressed genes in the TCGA and CGGA cohorts and discovered three small molecules, fedratinib (TG-101348), XMD-892, and MMPX, overlap between L1000FWD and CMap (Fig. 6C). Among them, fedratinib is an orally bioavailable, small molecule, ATP-competitive inhibitor of Janus-associated kinase 2 (JAK2). CCK-8 assay results showed that 2, 4, 6, 8, 10, and 25 μM of fedratinib inhibited the LGG cell viability, including SW1783 and SW1088 cell lines. Furthermore, when the same dose of fedratinib was used to treat SW1783 and SW1088 cells, cell viability was decreased after 48 hours compared to 24 hours (Fig. 6D). Fedratinib similarly decreased glioblastoma cell viability in three different glioblastoma cell lines studied in CCK-8 assay (Additional file 2: Fig. S6A). Fedratinib inhibits the viability of glioma cells in a dose- and time-dependent manner. Next, we added different concentrations of fedratinib to glioma cells treated. The results showed that the area and intensity of colony formation of glioma cells (SW1783, SW1088, and LN18) decreased significantly with the addition of > 0.4 μM of fedratinib (Fig. 6E, F and Additional file 2: Figs. S6B, C). Fedratinib inhibits colony formation and proliferation of glioma cells.

## Discussion

With the advancement of genome-wide screening and next-generation sequencing methods, transcriptome profiling has made significant contributions to a variety of fields, particularly cancer research, including studies on cancer heterogeneity and advancement, differential gene expression analysis and biomarkers, immunotherapy, and treatment response, and so on [41]. Transcriptome profiling has recently proven to be helpful for glioma prediction, prognosis, and improved molecular classification [14, 42]. As a result, the use of transcriptome profiling to identify cancer biomarkers, gene signatures, and their aberrant expression affecting oncogenesis, as well as the discovery of molecular targets for anticancer therapies, is critical and may result in a paradigm shift away from histopathology and organisms-based understandings. In this study, we found that the mRNA expression of PRGs correlated with the WHO grades of LGGs, and identified pyroptotic genes and molecular subtypes that were significantly associated with the prognosis of LGGs. A novel risk signature was developed to predict LGG patients’ prognosis and reflect the clinicopathological features, biological processes, and immune activation associated with malignancy. Additionally, the small molecule inhibitor fedratinib was screened using differential gene expression profiles based on the high RiskScore value enrichment of LGG patients. Experiments in cell lines of WHO grade III astrocytoma (SW1783 and SW1088), glioblastoma (LN18 and A172), and glioblastoma multiforme (T98G) revealed that fedratinib all showed tumor-suppressive effects.

Pyroptosis is a sort of regulated cell death: cell swelling, lysis, and the release of inflammatory mediators (IL-1β and IL-18) in character. In the classical pyroptosis pathway, when certain inflammasomes (NLRP1, NLRP3, NLRC4, and AIM2) stimulate pyroptosis-related pattern recognition receptors, they recruit and activate CASP1 either directly or by recruiting the receptor protein ASC recruits and activates CASP1, which successively promotes the maturation of IL-1β and IL-18 precursors and cleaves GSDMD [10]. Among other pyroptosis pathways, activated CASP4/5 specifically cleaves GSDMD [43], as well as CASP8 and CASP9 by affecting CASP3, causing cleavage of GSDME [44, 45]. Subsequently, pyroptosis pathways mediated by cleavage of other GSDM family proteins were identified, including GSDMA, GSDMB, and GSDMC [24, 46, 47]. Based on the above-identified pyroptosis pathways, we selected the most critical 24 regulators for study and established a prognostic signature consisting of 3 pyroptosis-related signature genes. With a deeper understanding of PCD, some recent studies have discovered significant crosstalk between pyroptosis, necroptosis, and apoptosis [48], resulting in the concept of PANoptosis. Although pyroptosis, necroptosis, and apoptosis are all PCD pathways, they are not the sole explanation for PANoptosis, which is defined as an inflammatory PCD pathway activated by a specific trigger and regulated by the PANoptosome complex. Pyroptosis is an exciting modality of PCD that seems to share crosstalk with apoptosis. In the Liu J et al. study [49], IHC results showed that GSDMD expression was significantly higher in glioma tissues and correlated with higher glioma WHO grade. Our study also showed higher levels of GSDMD expression in WHO grade III LGG. However, in cells lacking GSDMD, CASP1 has been shown to activate CASP9 to engage the apoptotic program [50]. Therefore, whether our prognostic features have broader applicability in the context of PANoptosis remains to be further explored. We found higher expression levels for CASP1 and CASP3 in glioma cell lines than in HEB cells; two risk factors thought to play an important role in glioma. In the Tong L et al. study [51], CASP1 inactivation significantly decreases IL-18 and IL-1β proteins and leads to U87 Cell Death. CASP3 in dying glioma cells after radiotherapy mediates proangiogenic response [52]. In vitro, activation of CASP9 impairs glioma cell invasion and angiogenesis, and overexpression of CASP9 in combination with radiation has a synergistic effect on glioma invasion inhibition [53]. Interestingly, we think that the protective factor CASP9, which is highly expressed in LGG tissues, does not show consistent results at the cellular level. However, in a study by Li XY et al. [54], it was concluded that CASP9 is also a protective factor in glioblastoma. In the study, the mRNA and protein levels were demonstrated that many clinical samples’ expression of CASP9 was significantly upregulated in glioma. This may be due to tumor cells in vitro culture that does not fully simulate the in vivo growth environment.

Exogenously activated pyroptosis has been shown to trigger potent antitumor activity, including physical and chemical factors. A recent study has revealed that GSDME is required for radiation-induced pyroptosis in cancer cells via the CASP3 pathway and that its expression increases the sensitivity of radioresistant colorectal cancer cells [12]. Yang X et al. showed that cold atmospheric plasma effectively induces pyroptosis in cancer cells by activating mitochondrial pathways (JNK/cytochrome c/CASP9/CASP3) and cleaving GSDME [55]. Our colleague Hu L et al. previously demonstrated that the predicted palmitoylation sites C407 and C408 in the GSDME C-terminal domain were involved in the chemotherapeutic drug-induced pyroptosis of cancer cells [56]. Liu J et al. concluded that TMZ treatment induced pyroptosis in glioblastoma cells, and GSDMD expression increased time-dependent following TMZ treatment [49]. Therefore, for the data collection of LGG patients, we included only patients with complete survival follow-up information and who received radiotherapy or chemotherapy. To investigate the value of the clinical application, differentially expressed PRGs were used in combination with OS in LGG patients for analysis, and a pyroptosis-related prognostic gene signature was constructed to assess the prognostic risk of LGG patients. We confirmed the prognostic value of the prognostic signature constructed in LGG. First, our model showed excellent predictive efficiency (AUC = 0.712-0.883) in terms of survival of LGG patients at 1, 3, and 5 years. Second, we discovered that RiskScore value was substantially connected with standard clinicopathological features (IDH mutation status, WHO grade, and 1p/19q codeletion status) when we used a prognostic model to determine patients’ RiskScore value. In LGG patients, RiskScore was an independent predictor of OS. Finally, we demonstrate that RiskScore values accurately predict outcomes in LGG patients regardless of whether they receive radiotherapy or chemotherapy. Still, the predictive efficiency is higher in treated patients, possibly due to the activation of pyroptosis modality in gliomas by clinical treatment.

Pyroptosis was initially found in immune cells, but with the study of the GSDM superfamily, it was found that cleavage of gadermins by the caspase activated pyroptosis in tumor cells. Zhang et al. have discovered that GZMB from killer cytotoxic lymphocytes induces GSDME cleavage and activation within cancer cells, promoting tumor cell phagocytosis by TAMs and activating NK and CD8+ T cells [11]. Moreover, in animal tests, Zhou et al. established that GZMA generated by cytotoxic NK and T lymphocytes cleaves another gasdermin in cancer cells, GSDMB, resulting in tumor elimination [24]. In our study, GO and KEGG analysis results showed that immune-related biological processes and pathways were significantly enriched in the Risk-H group. Meanwhile, LGG patients in the Risk-H group had a more abundant infiltration of immune cells, such as macrophage M1, macrophage M2, NK cells, neutrophils, CD4+ T cells, and CD8+ T cells. Notably, Macrophages are highly plastic innate immune cells that differentiate into two extreme phenotypes, M1 (anti-tumor and the expression of proinflammatory cytokines) and M2 (pro-tumor and immunoregulatory functions). Tumor-associated macrophages are the most abundant inflammatory cells in “cold” tumors in gliomas and are closely associated with glioma progression, treatment, and prognosis [39]. Three regulated necrotic cell death modalities in macrophages, necroptosis, pyroptosis and parthanatos contribute to distinct pathologies [57]. Wang Q et al. induced pyroptosis in immune checkpoint blockade resistant tumors and found an increase in the number of macrophage M1 and showed significant tumor suppression in combination with anti-PD-1 treatment [40]. Therefore, we systematically analyzed the immune checkpoints and immunotherapy response and found that the expression of most of the immune checkpoints was upregulated in the Risk-H group and that immunotherapy may have a higher response rate in the Risk-H group. A part of the key pyroptosis regulators was enriched in the Risk-H group of LGG patients, especially the CASP family and GSDMD (Additional file 2: Fig. S2J); however, it is worth noting that further experiments are needed to prove whether PRGs directly cause the immune activation state shown.

In glioma, antibody-drug conjugate targeting EGFR combined with TMZ has shown positive results, although current targeted therapy alone has not significantly impacted survival [4]. Small molecule inhibitors of kinases such as MEK, BRAF, EGFR, ALK, and KRAS promoted GSDME cleavage by CASP3 and resulted in lung cancer and melanoma regression [58, 59]. The JAK-STAT signaling pathway showed the highest NES score in the Risk-H group in the study. This may indicate a critical role for the JAK-STAT pathway in the Risk-H group, so we used differential genes between the Risk-H and Risk-L groups to predict and screen small molecule inhibitors, finally focusing on the JAK2 kinase inhibitor fedratinib. Fedratinib is used to treat intermediate-grade 2 and high-risk primary and secondary myelofibrosis but has not found application in the treatment of glioma and has therefore been selected as a candidate for glioma therapy. When fedratinib is administered orally, it competes with wild-type JAK2 and mutated forms to bind ATP, resulting in inhibition of JAK2 activation and JAK-STAT pathway inhibition. Jin Y et al. used fedratinib to inhibit M2-microglia polarization in vitro and reduce non-small cell lung cancer brain metastasis in vivo [60]. SW1088 in LGG cell lines and LN18 in glioblastoma cell lines showed higher RiskScore values, as determined by prognostic gene signature-based risk stratification of glioma cell lines (Additional file 7: Table S6). In our study, we discovered that the JAK-STAT pathway is activated in samples with high RiskScore values, so it is considered that fedratinib may have a stronger inhibitory effect in these samples. The IC50 of fedratinib was measured on SW1078 and SW1088 cells (Fig. 6D) and it was found that fedratinib was more effective in inhibiting SW1783 than SW1088, which was inconsistent with our expected results. We found that fedratinib significantly induced glioma cell death in vitro (Additional file 2: Figs. S6D-H). As a next step, we will continue to explore the types of glioma cell death induced by Fedratinib. These findings may pave the way for developing novel therapeutic targets or treatment strategies for glioma.

In conclusion, our study identifies a novel prognostic marker signature consisting of 3 pyroptosis-related signature genes. Based on the prognostic signature, we can guide a personalized therapeutic pathway in LGG and may hopefully be used to guide clinicians in prognosis and immune status prediction for LGG patients. Of course, there are some limitations to this study. The transcriptomic data of LGGs we used were derived from the TCGA and CGGA databases. Future validation using an independent LGG cohort with complete clinical information and gene expression information is necessary. Additionally, postoperative LGG tissues from clinical patients can be collected, patients can be stratified based on the prognostic signature, and then the malignant phenotypes of patient-derived glioma cells to verify the clinical application prospects of the signature. Finally, the antitumor mechanism of fedratinib in glioma cells needs to be further explored. Glioma is a notoriously immunologically “cold” tumor, and neither targeted therapy nor immunotherapy seems to result in significant prolongation of OS. Pyroptosis is induced in tumor cells during radiotherapy or chemotherapy and can reverse the immunosuppressive microenvironment. However, how pyroptosis is induced in gliomas remains unclear, and whether pyroptosis can turn gliomas from “cold” to “hot.” Taken together, understanding the mechanism of pyroptosis in cancer will help develop new therapeutic strategies and clinical translation, for which our work provides the foundation.

## Conclusion

We performed a comprehensive analysis of PRGs in LGG and combined 3 signature genes to construct a prognostic signature. The prognostic signature has shown excellent efficiency, stability, and accuracy in predicting and classifying patients’ prognoses. The signature also indicated differences in the immune status of risk subgroups. In addition, we confirmed that Fedratinib inhibits the viability and proliferation of glioma cells. Taken together, our results assist in identifying novel biomarkers for predicting the clinical prognosis and immune status of LGG and provide a foundation for preclinical studies targeting glioma drug therapy.

## Supplementary Information

**Additional file 1: Table S1.** Clinical features of patients with lower-grade gliomas in the training and validation cohorts.

**Additional file 2: Fig. S1.** Somatic mutation and correlation analysis of 24 PRGs and WHO grade analysis. (A) The TCGA mutation landscape in 520 LGG patients. Each waterfall plot represented information about the mutations in each gene. The tumor mutation burden (TMB) was depicted in the barplot above. Independently, the right numbers represented the frequency of mutation. (B) Bar plot displaying CNV frequency of 24 PRGs in TCGA cohort. (C) PPI analysis of 24 PRGs from the STRING platform, with colors and circle sizes representing MCC scores calculated by Cytoscape software. (D) Correlation analysis of expression levels between 24 PRGs. (E-F) The expression levels of 24 PRGs in LGG with different WHO grades in TCGA and CGGA cohorts. *P < 0.05, **P < 0.01, and ***P < 0.001. **Fig. S2.** Consensus clustering analysis and validation of prognostic signature. (A) The cophenetic, dispersion, rss, and silhouette coefficients are related to the number of clusters k. The cophenetic coefficient is used to reflect the stability of the NMF cluster, while rss is used to reflect the model’s clustering performance. (B) During clusters k = 2, consensus map of NMF clustering in CGGA validation cohort. (C) The Kaplan-Meier curves for the two clusters are based on CGGA cohort. (D) Bar graphs were used to show the proportion of LGG molecular subtypes among the two pyroptosis-associated clusters. (E) The scattergrams of the RiskScore value (up) and survival status (down) of LGG patients in the CGGA cohorts. (F-G) PCA for the LGG patients of different risks to distinguish Risk-H group from Risk-L group in the TCGA and CGGA cohorts. (H-I) The Kaplan–Meier survival curve of the prognostic signature predicting the Risk-H and Risk-L groups in the mRNA array 301 and Rembrandt cohorts. (J) The heatmap shows the correlation between the expression levels of the 24 PRGs and the cluster, WHO grade, and RiskScore values. Transcriptomic data for the 24 PRGs were used in TPM format. **P < 0.01 and ***P < 0.001. **Fig. S3.** Relationship between the RiskScore value and clinical features. (A) The heatmap shows the expression levels of the 3 signature genes. The distribution of clinical characteristics and C1/2 subgroups was compared between the Risk-H and Risk-L groups in the CGGA cohort. (B) Distribution of RiskScore value in the TCGA cohort stratified by grade, IDH status, 1p/19q codel status, and C1/2 subgroups. (C) Distribution of RiskScore value in the CGGA cohort stratified by grade, IDH status, 1p/19q codel status, and C1/2 subgroups. (D) The Kaplan–Meier survival curve of the prognostic signature predicting the Risk-H and Risk-L groups in the CGGA cohorts. (E-F) The Kaplan-Meier survival curves predict the OS of the LGG patients receiving different treatment strategies in the CGGA cohorts. *P < 0.05, **P < 0.01, and ***P < 0.001. **Fig. S4.** Analysis of the abundance of immune cell infiltration. (A-B) Comparison of the abundance of innate and adaptive immune cell infiltration between two risk subgroups in the CGGA cohort. (C) The correlation between the expression of CASP1, CASP3, and CASP9 and immune cells in LGG from the TIMER database. The infiltration level of 6 immune cells was corrected considering the tumor purity. (D) The comparison of macrophage infiltration between two risk subgroups in LGG. The scores of the four different algorithms were taken from the TIMER database. (E) The abundance of 11 immune functions by ssGSEA algorithm in the Risk-H and Risk-L groups in the CGGA cohort. *P < 0.05, **P < 0.01, and ***P < 0.001. **Fig. S5.** Different immune microenvironment and immune checkpoint profiles of risk subgroups. (A) The association between RiskScore value and stromal score, immune score, ESTIMATE score, and tumor purity in the CGGA cohort. (B) The expression levels of 23 immune checkpoints profiles in LGG with different risk subgroups in the CGGA cohort. *P < 0.05, **P < 0.01, and ***P < 0.001. **Fig. S6.** Fedratinib’s impact on the viability and proliferation of glioblastoma cells. (A) An CCK-8 assay was used to evaluate the viability of LN18, T98G, and A172 cells under different concentrations of fedratinib (1, 2, 4, 6, 8, 10, and 25 μM). Cells were treated with the same drug dose for 24 and 48 hours, respectively. *P < 0.05, **P < 0.01, ***P < 0.001, and ****P < 0.0001 vs. control group. (B) A colony formation assay was performed to detect the LN18 cell colony formation ability. (C) Quantitative analysis of colony formation formed by LN18 cells was performed using ImageJ, followed by visualization performed using GraphPad Prism. *P < 0.05, and **P < 0.01 vs. control group. (D) Microscopic images of SW1783 cells treated with different concentrations of fedratinib (2, 6, and 10 μM) for 24 or 48 hours. (E) Microscopic images of SW1088 cells treated with different concentrations of fedratinib (2, 6, and 10 μM) for 24 or 48 hours. (F) Microscopic images of LN18 cells treated with different concentrations of fedratinib (2, 6, and 10 μM) for 24 or 48 hours. (G) Microscopic images of T98G cells treated with different concentrations of fedratinib (2, 6, and 10 μM) for 24 or 48 hours. (H) Microscopic images of A172 cells treated with different concentrations of fedratinib (2, 6, and 10 μM) for 24 or 48 hours.

**Additional file 3: Table S2.** Univariate and multivariate Cox regression analysis of RiskScore value and clinical features.

**Additional file 4: Table S3.** List of immune cell-related metagenes and immune function-related immunomodulators.

**Additional file 5: Table S4.** GSEA analysis results in Risk-H group.

**Additional file 6: Table S5.** Drug or small molecule compounds predicted using L1000FWD and CMap tools.

**Additional file 7: Table S6.** RiskScore values for glioma cell lines predicted based on signature genes expression.

## Data Availability

The data supporting this study’s findings are openly available, which can be retrieved from the TCGA-LGG dataset from TCGA (https://portal.gdc.cancer.gov/repository?facetTab=cases), mRNAseq_693, mRNAseq_325, and mRNA-array_301 datasets from CGGA (http://www.cgga.org.cn/download.jsp), and gene_tpm_2017-06-05_v8_brain_cortex and frontal_cortex_ba9 datasets from GTEx (https://www.gtexportal.org/home/datasets).

## Abbreviations

PCD: Programmed cell death
PRGs: Pyroptosis-related genes
LGG: Lower-grade glioma
TMZ: Temozolomide
TCGA: The cancer genome atlas
CGGA: Chinese glioma genome atlas
L1000FWD: L1000 fireworks display
CMap: The connectivity map
LASSO: Least absolute shrinkage and selection operator
RNA-seq: RNA sequencing
GTEx: Genotype-tissue expression
OS: Overall survival
CNV: Copy number alteration
NMF: Non-negative matrix factorization
FC: Fold change
FDR: False discovery rate
ROC: Receiver operating characteristic
IDH: Isocitrate dehydrogenase
1p/19q: Chromosome arms 1p and 19q
ssGSEA: Single sample gene set enrichment analysis
GO: Gene ontology
KEGG: Kyoto encyclopedia of genes and genomes
TNF: Tumor necrosis factor
TIDE: Tumor immune dysfunction and exclusion
GSEA: Gene set enrichment analysis
MSigDB: Molecular Signatures Database
ATCC: American type culture collection
DMEM: Dulbecco’s modified Eagle’s medium
FBS: Fetal bovine serum
qRT-PCR: Real-time quantitative PCR
OD: Optical density
ANOVA: Analysis of variance
PPI: Protein-protein interaction network analysis
WHO: World health organization
IHC: Immunohistochemistry
PCA: Principal component analysis
CCR: Cytokine and cytokine receptor
HLA: Human leukocyte antigen
MHC: Major histocompatibility complex
CR: Complete response
PR: Partial response
SD: Stable disease
PD: Progressive disease
HR: Hazard ratio
TMB: Tumor mutation burden
