# Supplementary material for: Systematic transcriptome profiling of pyroptosis related signature for predicting prognosis and immune landscape in lower grade glioma
Source: BMC Cancer. 2022 Aug 13;22:885. doi: 10.1186/s12885-022-09982-7 (PMC9375370; doi:10.1186/s12885-022-09982-7)
Supplement: Supplementary file 1 — Additional file 1: Table S1. Clinical features of patients with lower-grade gliomas in the training and validation cohorts. [file 12885_2022_9982_MOESM1_ESM.docx]

**Additional file 1: Table S1.** Clinical features of patients with lower-grade gliomas in the training and validation cohorts.

|  | **Training cohort** |  | **Validation cohort** | | | | |
| --- | --- | --- | --- | --- | --- | --- | --- |
| **Clinical Features** | **TCGA(n=413)** |  | **CGGA(n=494)** |  | **mRNA array 301(n=142)** |  | **Rembrandt(n=133)** |
| **Age** |  |  |  |  |  |  |  |
| <=45 | 245 (59.32%) |  | 364 (73.68%) |  | 105 (73.94%) |  | 68 (51.13%) |
| >45 | 168 (40.68%) |  | 130 (26.32%) |  | 35 (24.65%) |  | 61 (45.86%) |
| NA | 0 (0.00%) |  | 0 (0.00%) |  | 2 (1.41%) |  | 4 (3.01%) |
| **Gender** |  |  |  |  |  |  |  |
| Male | 225 (54.48%) |  | 285 (57.69%) |  | 77 (55.23%) |  | 68 (51.13%) |
| Female | 188 (45.52%) |  | 209 (42.31%) |  | 65 (45.77%) |  | 41 (30.83%) |
| NA | 0 (0.00%) |  | 0 (0.00%) |  | 0 (0.00%) |  | 24 (18.05%) |
| **Grade** |  |  |  |  |  |  |  |
| II | 196 (47.46%) |  | 226 (45.75%) |  | 91 (64.08%) |  | 62 (46.62%) |
| III | 217 (52.54%) |  | 268 (54.25%) |  | 51 (35.92%) |  | 54 (40.60%) |
| NA | 0 (0.00%) |  | 0 (0.00%) |  | 0 (0.00%) |  | 17 (12.78%) |
| **Histology** |  |  |  |  |  |  |  |
| A | 46 (11.14%) |  | 139 (28.14%) |  | 60 (42.25%) |  | 57 (42.86%) |
| AA | 99 (23.97%) |  | 185 (37.45%) |  | 32 (22.54%) |  | 36 (27.07%) |
| AO | 81 (19.61%) |  | 78 (15.79%) |  | 15 (10.56%) |  | 18 (13.53%) |
| AOA | 37 (8.96%) |  | 0 (0.00%) |  | 4 (2.82%) |  | 0 (0.00%) |
| O | 109 (26.39%) |  | 92 (18.62%) |  | 18 (12.68%) |  | 22 (16.54%) |
| OA | 41 (9.93%) |  | 0 (0.00%) |  | 13 (9.15%) |  | 0 (0.00%) |
| NA | 0 (0.00%) |  | 0 (0.00%) |  | 0 (0.00%) |  | 0 (0.00%) |
| **IDH mutation status** |  |  |  |  |  |  |  |
| Mutant | 338 (81.84%) |  | 374 (75.71%) |  | 94 (66.20%) |  | 0 (0.00%) |
| Wildtype | 75 (18.16%) |  | 120 (24.29%) |  | 47 (33.10%) |  | 0 (0.00%) |
| NA | 0 (0.00%) |  | 0 (0.00%) |  | 1 (0.70%) |  | 133 (100.00%) |
| **1p/19q codeletion status** | |  |  |  |  |  |  |
| Codel | 142 (34.38%) |  | 155 (31.38%) |  | 13 (9.15%) |  | 8 (6.02%) |
| Non-codel | 271 (65.62%) |  | 339 (68.62%) |  | 28 (19.72%) |  | 23 (17.29%) |
| NA | 0 (0.00%) |  | 0 (0.00%) |  | 101 (71.13%) |  | 102 (76.69%) |
| **Radiotherapy** |  |  |  |  |  |  |  |
| treated | 270 (65.38%) |  | 384 (77.73%) |  | 133 (93.66%) |  | 0 (0.00%) |
| un-treated | 143 (34.62%) |  | 110 (22.27%) |  | 9 (6.34%) |  | 0 (0.00%) |
| NA | 0 (0.00%) |  | 0 (0.00%) |  | 0 (0%) |  | 133 (100.00%) |
| **Chemotherapy** |  |  |  |  |  |  |  |
| treated | 251 (60.77%) |  | 317 (64.17%) |  | 68 (47.89%) |  | 0 (0.00%) |
| un-treated | 162 (39.23%) |  | 177 (35.83%) |  | 74 (52.11%) |  | 0 (0.00%) |
| NA | 0 (0.00%) |  | 0 (0.00%) |  | 0 (0.00%) |  | 133 (100.00%) |
|  |  |  |  |  |  |  |  |
