# Supplementary material for: Systematic transcriptome profiling of pyroptosis related signature for predicting prognosis and immune landscape in lower grade glioma
Source: BMC Cancer. 2022 Aug 13;22:885. doi: 10.1186/s12885-022-09982-7 (PMC9375370; doi:10.1186/s12885-022-09982-7)
Supplement: Supplementary file 2 — Additional file 2: Fig. S1. Somatic mutation and correlation analysis of 24 PRGs and WHO grade analysis. (A) The TCGA mutation landscape in 520 LGG patients. Each waterfall plot represented information about the mutations in each gene. The tumor mutation burden (TMB) was depicted in the barplot above. Independently, the right numbers represented the frequency of mutation. (B) Bar plot displaying CNV frequency of 24 PRGs in TCGA cohort. (C) PPI analysis of 24 PRGs from the STRING platform, with colors and circle sizes representing MCC scores calculated by Cytoscape software. (D) Correlation analysis of expression levels between 24 PRGs. (E-F) The expression levels of 24 PRGs in LGG with different WHO grades in TCGA and CGGA cohorts. *P < 0.05, **P < 0.01, and ***P < 0.001. Fig. S2. Consensus clustering analysis and validation of prognostic signature. (A) The cophenetic, dispersion, rss, and silhouette coefficients are related to the number of clusters k. The cophenetic coefficient is used to reflect the stability of the NMF cluster, while rss is used to reflect the model’s clustering performance. (B) During clusters k = 2, consensus map of NMF clustering in CGGA validation cohort. (C) The Kaplan-Meier curves for the two clusters are based on CGGA cohort. (D) Bar graphs were used to show the proportion of LGG molecular subtypes among the two pyroptosis-associated clusters. (E) The scattergrams of the RiskScore value (up) and survival status (down) of LGG patients in the CGGA cohorts. (F-G) PCA for the LGG patients of different risks to distinguish Risk-H group from Risk-L group in the TCGA and CGGA cohorts. (H-I) The Kaplan–Meier survival curve of the prognostic signature predicting the Risk-H and Risk-L groups in the mRNA array 301 and Rembrandt cohorts. (J) The heatmap shows the correlation between the expression levels of the 24 PRGs and the cluster, WHO grade, and RiskScore values. Transcriptomic data for the 24 PRGs were used in TPM format. **P < 0.01 and ***P < [file 12885_2022_9982_MOESM2_ESM.docx]

Additional file 2

**Title**

Systematic transcriptome profiling of pyroptosis related signature for predicting prognosis and immune landscape in lower grade glioma

**Author Information**

Huihan Yu^1,2,3,#^, Meiting Gong^1,2,3,#^, Jian Qi^2^, Wanxiang Niu^2^, Chenggang Zhao^2^, Suling Sun^2^, Shuyang Li^1,2^, Bo Hong^2,3^, Junchao Qian^2,3^, Hongzhi Wang^1,2,3,*^, Xueran Chen^2,3,*^, Zhiyou Fang^1,2,3,*^

^1^School of Basic Medical Sciences, Anhui Medical University, No. 81, Meishan Road, Hefei , Anhui , 230032, China

^2^Anhui Province Key Laboratory of Medical Physics and Technology; Institute of Health and Medical Technology, Hefei Institutes of Physical Science, Chinese Academy of Sciences, No. 350, Shushan Hu Road, Hefei, Anhui, 230031, China

^3^Department of Laboratory Medicine, Hefei Cancer Hospital, Chinese Academy of Sciences, No. 350, Shushan Hu Road, Hefei, Anhui, 230031, China

^#^ These authors contributed equally to this work.

*Corresponding author: Prof. Hongzhi Wang (wanghz@hfcas.ac.cn), Dr. Xueran Chen ([xueranchen@cmpt.ac.cn](mailto:xueranchen@cmpt.ac.cn)) and Zhiyou Fang ([zyfang@cmpt.ac.cn](mailto:zyfang@cmpt.ac.cn)).

**Additional file 2 includes:**

**Figure S1-S6.**

**
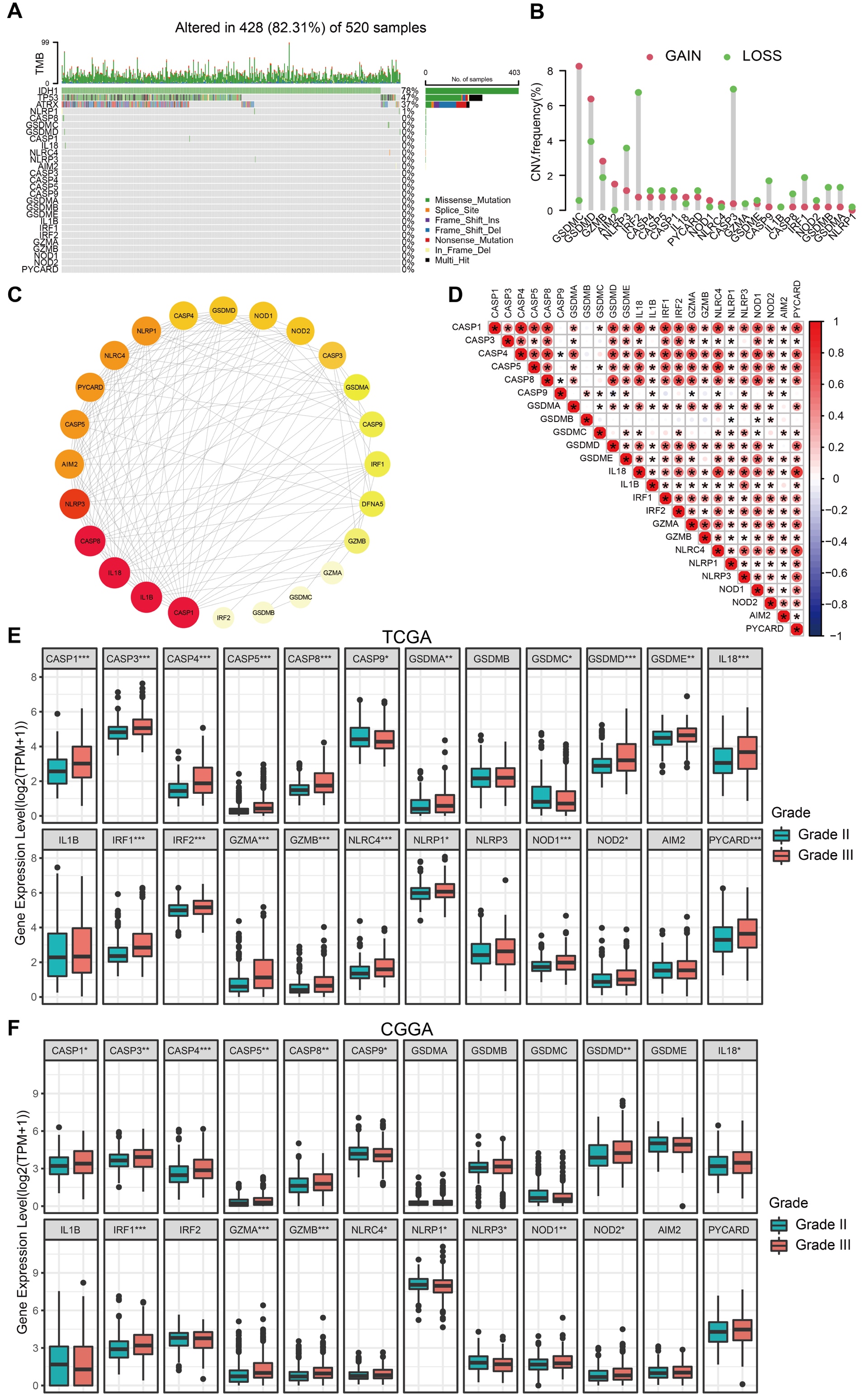
**

**Additional file 2: Figure S1.** **Somatic mutation and correlation analysis of 24 PRGs and WHO grade analysis.**

(**A**) The TCGA mutation landscape in 520 LGG patients. Each waterfall plot represented information about the mutations in each gene. The tumor mutation burden (TMB) was depicted in the barplot above. Independently, the right numbers represented the frequency of mutation. (**B**) Bar plot displaying CNV frequency of 24 PRGs in TCGA cohort. (**C**) PPI analysis of 24 PRGs from the STRING platform, with colors and circle sizes representing MCC scores calculated by Cytoscape software. (**D**) Correlation analysis of expression levels between 24 PRGs. (**E-F**) The expression levels of 24 PRGs in LGG with different WHO grades in TCGA and CGGA cohorts. *P < 0.05, **P < 0.01, and ***P < 0.001

**
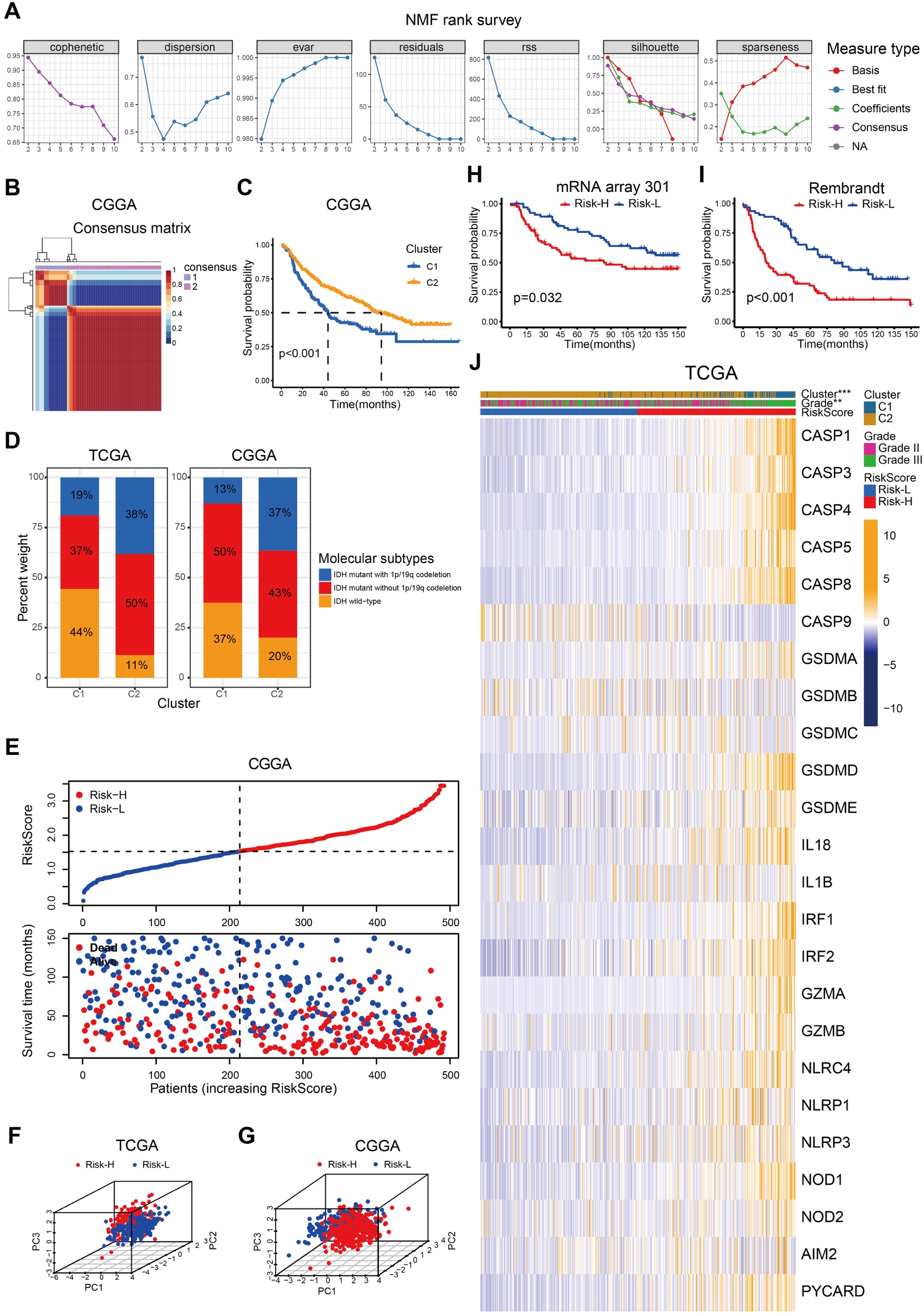
**

**Additional file 2: Figure S2.** **Consensus clustering analysis and validation of prognostic signature.**

(**A**) The cophenetic, dispersion, rss, and silhouette coefficients are related to the number of clusters k. The cophenetic coefficient is used to reflect the stability of the NMF cluster, while rss is used to reflect the model's clustering performance. (**B**) During clusters k = 2, consensus map of NMF clustering in CGGA validation cohort. (**C**) The Kaplan-Meier curves for the two clusters are based on CGGA cohort. (**D**) Bar graphs were used to show the proportion of LGG molecular subtypes among the two pyroptosis-associated clusters. (**E**) The scattergrams of the RiskScore value (up) and survival status (down) of LGG patients in the CGGA cohorts. (**F-G**) PCA for the LGG patients of different risks to distinguish Risk-H group from Risk-L group in the TCGA and CGGA cohorts. (**H-I**) The Kaplan–Meier survival curve of the prognostic signature predicting the Risk-H and Risk-L groups in the mRNA array 301 and Rembrandt cohorts. (**J**) The heatmap shows the correlation between the expression levels of the 24 PRGs and the cluster, WHO grade, and RiskScore values. transcriptomic data for the 24 PRGs were used in TPM format. **P < 0.01 and ***P < 0.001

**
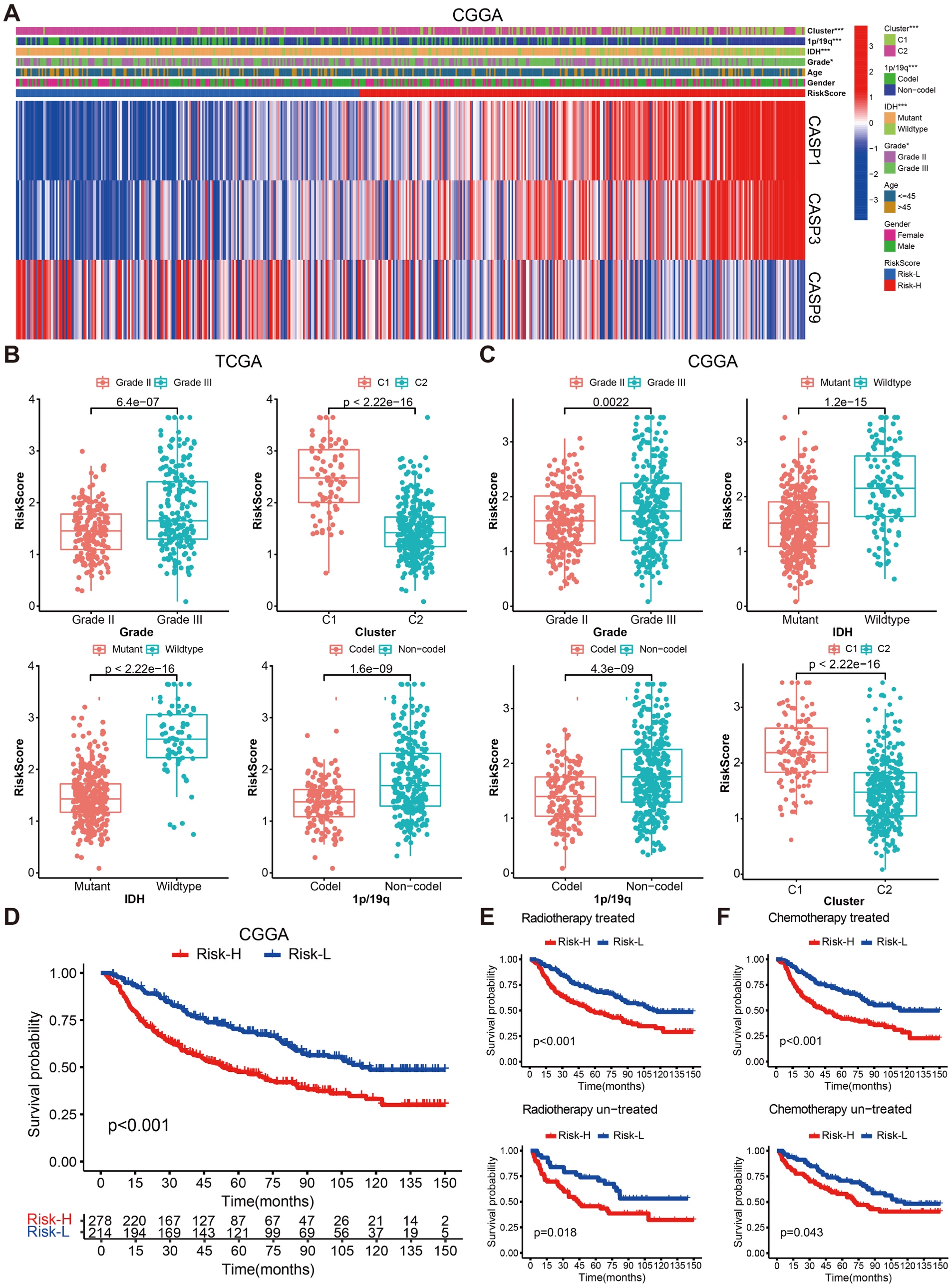
**

**Additional file 2: Figure S3.** **Relationship between the RiskScore value and clinical features.**

(**A**) The heatmap shows the expression levels of the 3 signature genes. The distribution of clinical characteristics and C1/2 subgroups was compared between the Risk-H and Risk-L groups in the CGGA cohort. (**B**) Distribution of RiskScore value in the TCGA cohort stratified by grade, IDH status, 1p/19q codel status, and C1/2 subgroups. (**C**) Distribution of RiskScore value in the CGGA cohort stratified by grade, IDH status, 1p/19q codel status, and C1/2 subgroups. (**D**) The Kaplan–Meier survival curve of the prognostic signature predicting the Risk-H and Risk-L groups in the CGGA cohorts. (**E-F**) The Kaplan-Meier survival curves predict the OS of the LGG patients receiving different treatment strategies in the CGGA cohorts. *P < 0.05, **P < 0.01, and ***P < 0.001


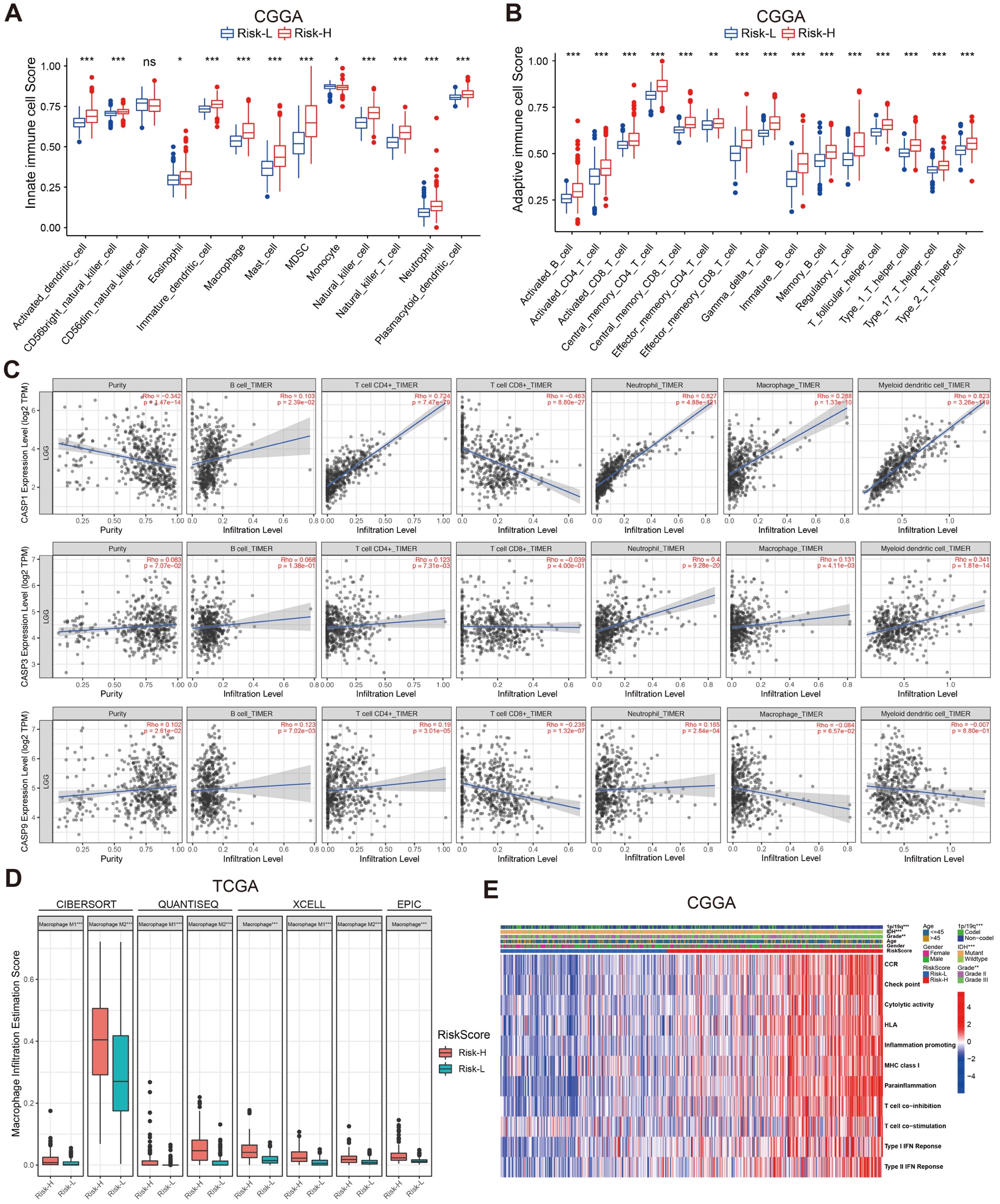


**Additional file 2: Figure S4.** **Analysis of the abundance of immune cell infiltration.**

(**A-B**) Comparison of the abundance of innate and adaptive immune cell infiltration between two risk subgroups in the CGGA cohort. (**C**) The correlation between the expression of CASP1, CASP3, and CASP9 and immune cells in LGG from the TIMER database. The infiltration level of 6 immune cells was corrected considering the tumor purity. (**D**) The comparison of macrophage infiltration between two risk subgroups in LGG. The scores of the four different algorithms were taken from the TIMER database. (**E**) The abundance of 11 immune functions by ssGSEA algorithm in the Risk-H and Risk-L groups in the CGGA cohort. *P < 0.05, **P < 0.01, and ***P < 0.001


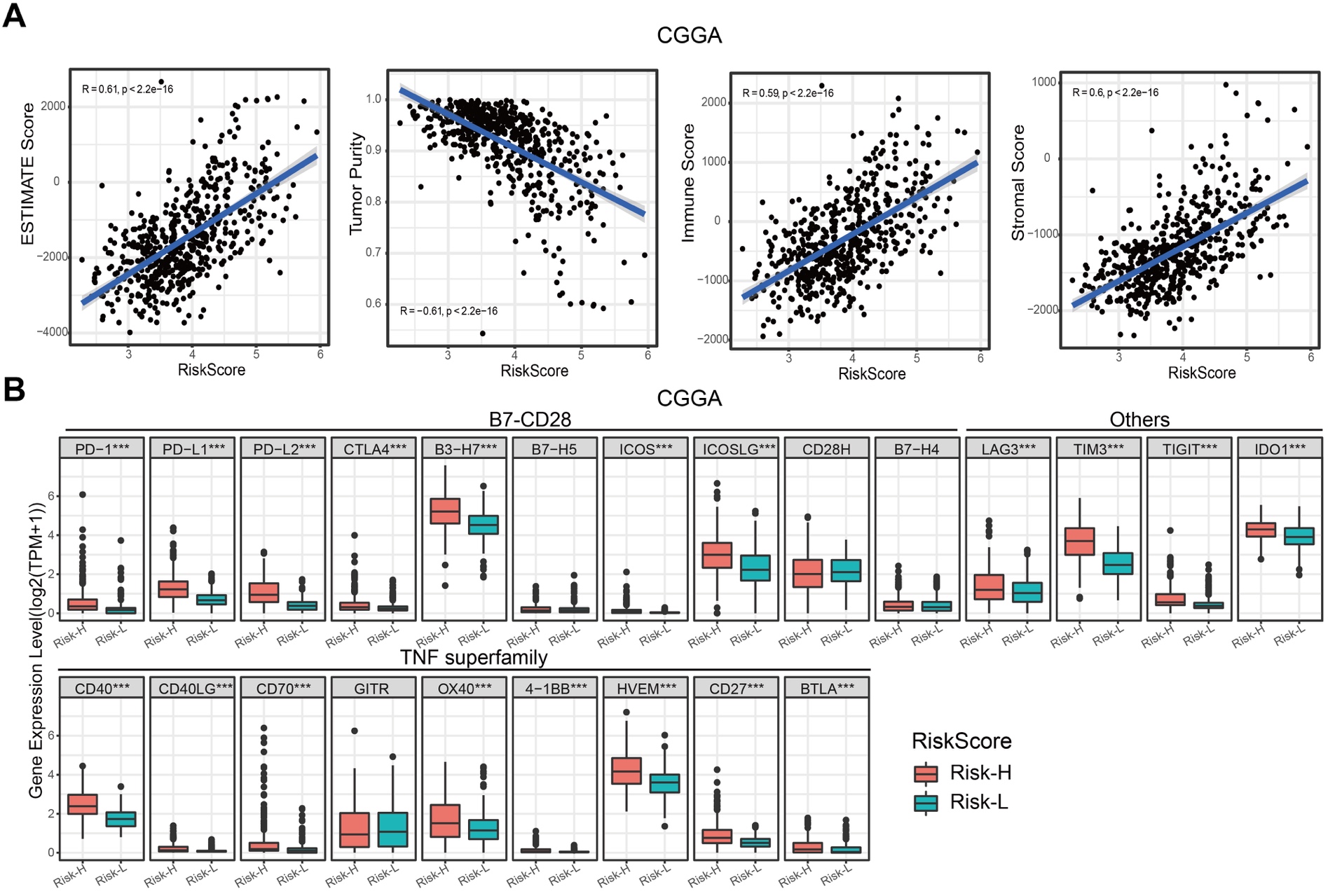


**Additional file 2: Figure S5.** **Different immune microenvironment and immune checkpoint profiles of risk subgroups.**

(**A**) The association between RiskScore value and stromal score, immune score, ESTIMATE score, and tumor purity in the CGGA cohort. (**B**) The expression levels of 23 immune checkpoints profiles in LGG with different risk subgroups in the CGGA cohort. *P < 0.05, **P < 0.01, and ***P < 0.001


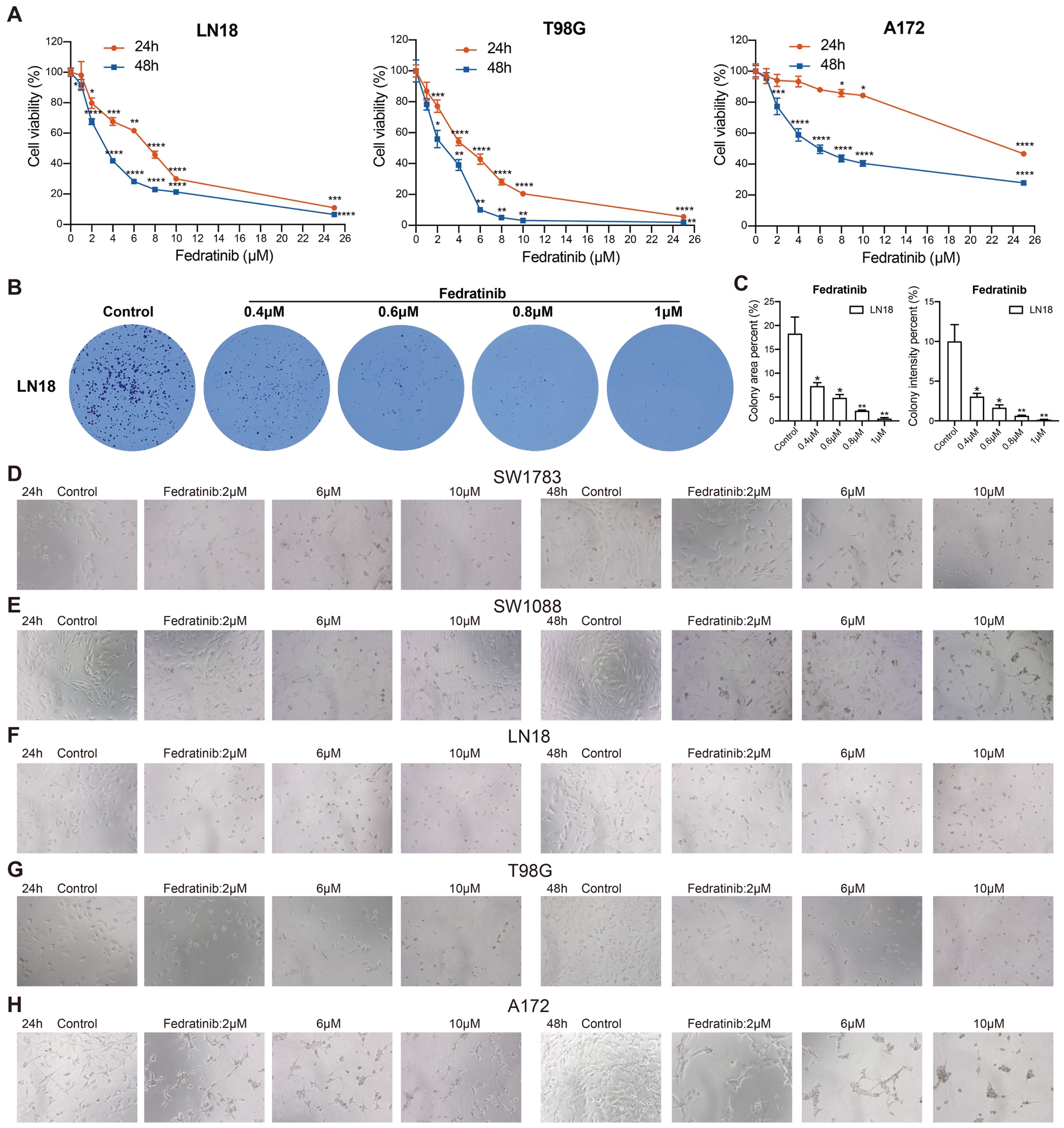


**Additional file 2: Figure S6.** **Fedratinib's impact on the viability and proliferation of glioblastoma cells.**

(**A**) An CCK-8 assay was used to evaluate the viability of LN18, T98G, and A172 cells under different concentrations of fedratinib (1, 2, 4, 6, 8, 10, and 25μM). Cells were treated with the same drug dose for 24 and 48 hours, respectively. *P < 0.05, **P < 0.01, ***P < 0.001, and ****P < 0.0001 vs. control group. (**B**) A colony formation assay was performed to detect the LN18 cell colony formation ability. (**C**) Quantitative analysis of colony formation formed by LN18 cells was performed using ImageJ, followed by visualization performed using GraphPad Prism. *P < 0.05, and **P < 0.01 vs. control group. (**D**) Microscopic images of SW1783 cells treated with different concentrations of fedratinib (2, 6, and 10μM) for 24 or 48 hours. (**E**) Microscopic images of SW1088 cells treated with different concentrations of fedratinib (2, 6, and 10μM) for 24 or 48 hours. (**F**) Microscopic images of LN18 cells treated with different concentrations of fedratinib (2, 6, and 10μM) for 24 or 48 hours. (**G**) Microscopic images of T98G cells treated with different concentrations of fedratinib (2, 6, and 10μM) for 24 or 48 hours. (**H**) Microscopic images of A172 cells treated with different concentrations of fedratinib (2, 6, and 10μM) for 24 or 48 hours.
