# Supplementary material for: Systematic transcriptome profiling of pyroptosis related signature for predicting prognosis and immune landscape in lower grade glioma
Source: BMC Cancer. 2022 Aug 13;22:885. doi: 10.1186/s12885-022-09982-7 (PMC9375370; doi:10.1186/s12885-022-09982-7)
Supplement: Supplementary file 3 — Additional file 3: Table S2. Univariate and multivariate Cox regression analysis of RiskScore value and clinical features. [file 12885_2022_9982_MOESM3_ESM.docx]

**Additional file 3: Table S2.** Univariate and multivariate Cox regression analysis of RiskScore value and clinical features.

|  |  |  | **TCGA** | | |  |  |  |  |  | **CGGA** | | |  |  |
| --- | --- | --- | --- | --- | --- | --- | --- | --- | --- | --- | --- | --- | --- | --- | --- |
|  | **Univariate** | | |  | **Multivariate** | | |  | **Univariate** | | |  | **Multivariate** | | |
| **Clinical Features** | **HR** | **95% Cl** | **P-value** |  | **HR** | **95% Cl** | **P-value** |  | **HR** | **95% Cl** | **P-value** |  | **HR** | **95% Cl** | **P-value** |
| Age | 1.055 | 1.038−1.072 | <0.001 |  | 1.056 | 1.037−1.075 | <0.001 |  | 1.017 | 1.004−1.030 | 0.011 |  | 1.014 | 1.002−1.026 | 0.022 |
| Gender | 1.091 | 0.731−1.630 | 0.669 |  |  |  |  |  | 1.012 | 0.784−1.306 | 0.925 |  |  |  |  |
| Grade | 3.252 | 2.077−5.092 | <0.001 |  | 1.717 | 1.056−2.791 | 0.029 |  | 3.347 | 2.525−4.435 | <0.001 |  | 3.084 | 2.311−4.115 | <0.001 |
| IDH mutation status | 0.144 | 0.095−0.217 | <0.001 |  | 0.585 | 0.309−1.108 | 0.1 |  | 0.438 | 0.335−0.575 | <0.001 |  | 0.841 | 0.619−1.141 | 0.266 |
| 1p/19q codeletion status | 0.364 | 0.218−0.607 | <0.001 |  | 0.525 | 0.292−0.943 | 0.031 |  | 0.282 | 0.201−0.396 | <0.001 |  | 0.34 | 0.238−0.487 | <0.001 |
| RiskScore | 3.367 | 2.587−4.383 | <0.001 |  | 2.045 | 1.415−2.957 | <0.001 |  | 2.26 | 1.866−2.737 | <0.001 |  | 1.631 | 1.335−1.993 | <0.001 |
| **Abbreviations: HR, Hazard ratio. Cl, Confidence interval.** | | |  |  |  |  |  |  |  |  |  |  |  |  |  |
